# Variability and methodological choices in articulatory suppression tasks: a review

**DOI:** 10.3389/fpsyg.2026.1736170

**Published:** 2026-03-18

**Authors:** Chiara De Livio, Angelo Mattia Gervasi, Ilenia Falcinelli, Chiara Fini, Fernando Maggio, Claudio Brozzoli, Anna M. Borghi

**Affiliations:** 1 Department of Psychology, Sapienza University of Rome, Rome, Italy; 2 ImpAct team, Lyon Neuroscience Research Center, INSERM, Bron, France; 3 Université Claude Bernard Lyon 1, Villeurbanne, France; 4 Department of Dynamic and Clinical Psychology, and Health Studies, Sapienza University of Rome, Rome, Italy; 5 Institute of Cognitive Sciences and Technologies, Italian National Research Council, Rome, Italy

**Keywords:** articulatory suppression, dual-task paradigm, embodied cognition, language, linguistic interference

## Abstract

Researchers often examine the role of overt language and inner speech in cognition by using articulatory suppression as a form of verbal interference. The hypothesis behind is that it causally prevents participants from using verbal strategies during the execution of a primary task. Articulatory suppression involves uttering either meaningful or meaningless syllables or words––aloud, silently, whispered. Although widely used to disrupt linguistic processing, the heterogeneity of procedures raises questions about the specificity of its effects, and its underlying mechanisms remain unclear. Studies vary in verbal stimuli, articulation modalities, rhythms, and modes of stimulus presentation. However, there are few empirical investigations about the impact of these methodological differences on the interference effects. Articulatory suppression also has different purposes: it is employed as the main experimental manipulation or as a control task. When it is the primary manipulation, once the suppression methods have been identified, attention should shift toward determining the most appropriate dual-task control condition. Conversely, when articulatory suppression is used as a control task, methodological details are often insufficiently reported, limiting replicability. This mini-review examines articles from the last decade that use articulatory suppression to disrupt phonological access during the execution of a primary task. We provide an overview of protocols employing articulatory suppression, emphasizing the heterogeneity of methodological choices and discussing the implications of this heterogeneity within embodied cognition. We stress the need for evidence on the neurocognitive implications of different articulatory suppression modalities. This would improve awareness when choosing modalities, consequently enhancing coherence across studies and replicability.

## 1 Introduction

### 1.1 Theoretical assumptions underlying articulatory suppression

The use of dual-task methodologies involving verbal interference is grounded in evidence from various research domains showing that language supports cognition (Alderson-Day and Fernyhough, 2015; Baddeley et al., 2001). Engaging the same cognitive resources—for example, by continuously repeating a syllable—can impair performance on a concurrent primary task. Researchers often examine the role of overt and covert language in cognition using verbal interference as a secondary, concomitant task during the execution of the primary one, which is hypothesized to involve language (Banks and Connell, 2024). If performance on the primary task is significantly impacted by linguistic interference, language is supposed to play an effective role in the primary task. Also, when studying visual working memory, researchers use linguistic interference as a control condition to prevent participants from using verbal instructions to complete the task (Brady and Störmer, 2022). Blocking possible verbal rehearsal strategies allows researchers to specifically investigate and isolate the visual working memory processes implied in the task. In both these cases, the most used verbal interference task in the cognitive sciences is articulatory suppression (Nedergaard et al., 2023, for a review). While its use is well-established, the wide variety of articulatory suppression tasks employed in research suggests that choosing the most appropriate one is not always straightforward. Articulatory suppression is well established as a means to causally disrupt the use of inner speech by involving the mouth motor components to produce sounds, consequently disabling the articulatory control system of the phonological loop (Larsen and Baddeley, 2003). The articulatory control system allows individuals to mentally rehearse key verbal information to maintain it in working memory (Baddeley and Hitch, 2019). A good example of this is when people receive a security code via text message and have to remember it for a few seconds to complete an online payment. The articulatory suppression system allows them to mentally articulate and maintain that code in working memory. By asking participants to perform articulatory suppression, researchers essentially disable this process, either totally or to a significant degree. Thus, for a long time, articulatory suppression has been investigated primarily as a cognitive task that interferes with working memory (Baddeley et al., 1984). However, articulatory suppression, regardless of the conveyed meaning, impacts conceptual processing/production, highlighting the involvement of the speech motor intention. Importantly, there is evidence that impairing linguistic articulation plays a causal role in comprehension and recall (D'Ausilio et al., 2009), highlighting the tight relationship between language production and comprehension.

### 1.2 Articulatory suppression in the framework of embodied cognition

Recent debates on the future of embodied cognition have highlighted the need for paradigms whose results cannot be easily interpreted or accommodated within other frameworks. Namely, sometimes embodied cognition studies do not allow for “strong inference,” i.e., they do not provide decisive evidence for one hypothesis over competing ones, and fail to draw causal conclusions.

Interference paradigms with healthy participants can provide opportunities to test hypotheses directly derived from an embodied perspective (Ostarek and Huetting, 2019; Ostarek and Bottini, 2021). In this framework, investigating articulatory suppression, which is thought to interfere with inner speech, can play a critical role in providing evidence in favor of embodiment. Importantly, however, studies on inner speech have yielded two contrasting views: according to the so-called “abstraction view” (e.g., Jones, 2009), inner speech involves only the initial stages of speech production before articulation; it is therefore more condensed and does not involve bodily actions. In contrast, the motor simulation view (Loevenbruck et al., 2018) underlines the importance of articulation for inner speech; this view relies, for example, on EMG evidence, which is, however, not always consistent (e.g., Nalborczyk et al., 2020). Notably, we do not believe that even the first view is incompatible with an embodied and grounded view of semantics (Reilly et al., 2023): covertly pronouncing the word “hand” may activate the hand motor system, even if lips or tongue are not directly involved in the word’s articulation (Borghi, 2023). Interesting approaches combine the two views, suggesting that inner speech varies along a continuum from featural richness to abstraction (Alderson-Day and Fernyhough, 2015; Fernyhough and Borghi, 2023). Indeed, some forms of inner speech are more condensed (Vygotsky, 1934/1987), thus not fully articulated, and therefore less detectable in their embodied components. In this regard, studies on articulatory suppression can be particularly informative for embodied cognition, since they help determine when and how motor simulation and articulation are involved during cognitive processing.

### 1.3 Current work

This mini-review collects and examines studies employing articulatory suppression, providing an overview of its different purposes and modalities. To facilitate future investigations and provide a comprehensive overview of articulatory suppression paradigms, we summarize the studies published over the past 10 years, detailing the key features of the methodology used.

We report methodological choices along the main dimensions of dual-task paradigms involving articulatory suppression and highlight the substantial heterogeneity in the ways this task is applied across studies.

Through a targeted review over the last decade of research employing articulatory suppression to induce verbal interference, we underscore the absence of validated, and cross-linguistically generalizable standards guiding its use. In doing so, we draw attention to the “garden of forking paths” characterizing its implementation, and highlight the need for researchers to be more aware of the consequences of the different methodological choices, suggesting more rigorous methodologies in the use of this task, and more detailed reporting of task design features to enhance transparency and replicability in science.

### 1.4 Method

We conducted a literature search of journal articles on Google Scholar between August and October 2025. We inserted the keywords “articulatory suppression” – “clinical” – “developmental” – “brain imaging.” Articles were excluded if they were inaccessible, not peer-reviewed, or not written in English. To refine the dataset, we applied the following criteria: inclusion criteria were studies reporting empirical behavioral data on articulatory suppression using behavioral paradigms; exclusion criteria included reviews, meta-analyses, conference abstracts. The screening process involved reviewing titles and abstracts for relevance, followed by full-text examination. The final corpus comprised 147 peer-reviewed studies. Studies were classified depending on whether articulatory suppression constituted the primary experimental manipulation (Supplementary Table 1) or the control condition (Supplementary Table 2). For each study, we extracted key methodological information on the articulatory suppression paradigm, including whether articulatory suppression was the main experimental manipulation, the type of control condition, the stimulus used, its modality, rhythm, monitoring procedures, and presentation format, and the language of the articulatory suppression stimulus materials. We also recorded information on the primary task in which articulatory suppression was applied, including its dependent variable, the stimulus used, as well as reporting the authors and year of publication.

## 2 A garden of forking paths: methodological variability in articulatory suppression research

Articulatory suppression involves uttering either syllables, phonemes, or words across different modalities––aloud, silently, whispered––or even silent mouth movements. These stimuli are usually presented either auditorily or visually to participants, who are required to repeat them continuously at a specific rhythm or at a self-paced rate. Although articulatory suppression is widely used to disrupt language processing, the cognitive and motor mechanisms through which different forms of articulatory suppression exert their effects remain unclear. This leaves open important questions about whether variations such as open versus close vowels, meaningful versus meaningless monosyllables, or overt versus covert articulation engage distinct processes and, consequently, produce different patterns of interference.

We identify five factors that vary in the implementation of articulatory suppression and that modulate inner speech along a continuum ranging from more embodied to more abstract forms: (i) the articulated stimuli; (ii) the modalities of the articulatory suppression; (iii) the rhythm of articulation; (iv) the presentation format of the stimuli; (v) cognitive load.

### 2.1 Articulated stimuli

Current research employs various phonemes, syllables, and words (see Supplementary Table 1 and Supplementary Table 2). These materials vary across two main dimensions: *articulatory complexity* and *lexical status*.

#### 2.1.1 Articulatory complexity and lexical status

Stimuli materials differ across studies in the complexity of articulatory demands. Regarding length, stimuli range from single syllables (e.g., *la*) to multi-syllabic words (e.g., *December*) and numbers, which can be either single digits (e.g., *3*) or multi-digit sequences (e.g., *753*). The stimuli also differ in meaningfulness: meaningless stimuli include syllables without semantic content (e.g., *ba*) and nonwords (e.g., *BABATAKA*), whereas *meaningful* stimuli include syllables with lexical value (e.g., *the*), full words (e.g., *California*), and numbers, whose meaning depends on the context. In most cases, the choice of stimuli is not justified by explicit predictions about the expected effect of interference on the primary task. Both theoretical models of speech production (Levelt, 1992, Levelt et al., 1999) and empirical studies (Bohland and Guenther, 2006; Riecker et al., 2008) indicate that increasing syllabic or sequential complexity places greater demands on the speech production system. Evidence further suggests that syllable- or word-sized units can be learned and stored as articulatory “chunks,” forming a *mental syllabary* that enables frequently used forms to be executed efficiently as single motor programs (Levelt and Wheeldon, 1994). In contrast, less practiced or longer words must be assembled online from multiple subunits, thereby increasing articulatory load and planning time (Tomaschek et al., 2021). Consistent with this view, a syllable frequency effect has been observed in speech production (Cholin et al., 2006), indicating that articulatory routines become automatized through repeated use. In addition, articulatory complexity varies systematically across phonetic and phonological structures (Locke, 1972; Glanz et al., 2022; Shuster and Cottrill, 2015; Ziegler and Aichert, 2015), reinforcing the idea that articulation efficiency depends jointly on word length, structural complexity, and motor familiarity. This is reflected in longer motor plan durations, increased articulator variability, and delays in onset time even in fluent speakers. Interestingly, while articulatory suppression has been used to investigate the effect of long vs. short words on recall (Romani et al., 2005; Russo and Grammatopoulou, 2003), no study has tackled whether using long vs. short stimuli in the articulatory suppression may have a different impact. At the same time, asking participants to repeat meaningful words (e.g., *Racket* or *Monday*) could reactivate different semantic contents related to those words. The same would not happen when asking participants to repeat a meaningful syllable (e.g., *the*), which has no meaning in the context of the articulatory suppression task (as for function words), or a meaningless syllable (e.g., *ta*). Consequently, the different semantic loads associated with the materials used for articulatory suppression must be carefully considered, as they may engage cognitive processes beyond mere articulation. Indeed, experimental evidence from healthy participants demonstrates that the semantic and syntactic properties of words become available slightly before their phonological forms during speech production (Indefrey and Levelt, 2004; Jescheniak et al., 2002), suggesting that meaningful stimuli could trigger pre-phonological lexical–semantic activation, potentially influencing task performance.

Notably, the studies summarized in Supplementary Table 1 and Supplementary Table 2 also differ in the language used for the articulatory suppression stimuli, and in several cases, neither the language nor participants’ native-speaker status was reported, which poses challenges for assessing cross-study comparability and potential language-specific effects.

### 2.2 Modalities of articulatory suppression

Verbal interference strategies also vary in the modality of the articulation employed. Stimuli may be articulated aloud, whispered, mouthed silently, or repeated covertly (i.e., subvocally). These variations in articulation modality may differentially engage cognitive and motor resources, potentially influencing the degree of interference produced. For instance, in overt articulation, speaking generates auditory and somatosensory feedback (Franken et al., 2022; Orepic et al., 2023) that participants inevitably process, whereas this feedback is partially or totally absent during covert or silent articulation. In the cases of silent articulation—mouthed or subvocal—it is not possible for the experimenter to externally verify whether the task is performed correctly. Reporting both the articulation modalities and any procedure used to monitor performance can therefore provide important methodological context and enhance the reproducibility of results.

### 2.3 Rhythm of articulation

An even less explored aspect of articulatory suppression is the rhythm at which the verbal material is repeated. The required repetition rate varies considerably across studies, and no standardized benchmarks exist for determining an optimal frequency. This lack of consistency complicates comparisons between studies and may influence the strength of the interference effect. The major difference is between paradigms with self-paced articulatory suppression and others with an imposed one. Even in the second case, different articulation frequencies may be requested from participants. This, in turn, raises more questions about whether and how the experimenter monitors these rhythms during the execution. In some cases, even if rarely, a metronome is employed, and participants are facilitated in keeping the rhythm (van ‘t Wout and Jarold, 2022). However, including a metronome adds auditory stimulation to the task ensemble.

### 2.4 Presentation format of the stimuli

After reviewing several methodological dimensions of articulatory suppression that might impact its effectiveness as a dual-task condition, we now turn to the manner in which the stimuli are usually delivered.

The component responsible for verbal working memory is the phonological loop, which is typically divided into two components: the phonological store and the articulatory control system. Auditory stimuli can be directly encoded into the phonological store, whereas visually presented material must first be recorded via the phonological loop before entering the articulatory control system (Baddeley and Hitch, 2019). Different studies variably employ auditory or visual (written) presentation of stimuli in articulatory suppression tasks (see Supplementary Table 1 and Supplementary Table 2). Notably, the modality of presentation often differs across the primary task, the suppression task, and the control task—sometimes inconsistently. This inconsistency becomes particularly relevant when analyzing phonological similarity effects, as the underlying mechanisms differ depending on input modality: phonological similarity tends to impair performance with visually presented materials but may facilitate processing in auditory contexts. Empirical findings (Baddeley et al., 1984; Macken et al., 2014) show that articulatory suppression typically abolishes the phonological similarity effect when phoneme lists are presented visually, whereas the effect often persists with auditory presentation. Indeed, the mechanisms proposed to underlie phonological similarity effects vary depending on whether the stimulus materials are presented visually or auditorily, with evidence of compromised retrieval under verbal interference conditions—particularly when visually presented materials are paired with phonologically dissimilar items (Nedergaard et al., 2023).

### 2.5 Cognitive load

Asking participants to repeat a sequence of multiple syllables such as *ba-be-bi-bo-bu* rather than a unique meaningful syllable, such as *the*, and to repeat them at variable frequencies, adds a further element to the picture, the cognitive load. Here, we refer to the cognitive load as the amount of working memory resources that the participants are required to imply when asked to repeat a combination of syllables or numbers, rather than a single syllable or digit, at a certain frequency. Maintaining in working memory a three-digit number (e.g., *753*) instead of a single-digit number, for instance, increases the amount of cognitive resources implied by the participant in the secondary task. In addition, repeating the syllables at a frequency of 2 Hz rather than 1 Hz implies a higher amount of cognitive resources. Therefore, these different choices lead to differences in terms of cognitive load that must be carefully considered when designing and interpreting such tasks.

## 3 The role of the articulatory suppression in the experimental design

Examining articles employing articulatory suppression, it emerges that there are two main reasons why authors adopt this paradigm. First, they use it as a main experimental manipulation to directly investigate whether language plays a role in a given task. Second, they employ it as a control task to isolate the contribution of a non-linguistic cognitive function in a task where language may still be recruited—for example, in visual working memory tasks. In the next two sections, we illustrate both possibilities of use of the articulatory suppression.

In the framework of studies investigating the role of language in cognition, researchers address whether language is involved in specific tasks, whether linguistic or not. In this context, articulatory suppression plays a crucial role in the experimental design, being the main manipulation condition. If language is effectively involved in the primary task, the inability to access it due to the interference should lead to an impairment in the performance (Banks and Connell, 2024). Once the role of the articulatory suppression in the experimental design has been identified, it is critical for authors to determine the most appropriate dual-task control condition. As suggested by Emerson and Miyake (2003), a passive control condition is typically insufficient to ensure proper experimental control. Considering that performing a simultaneous secondary task impairs performance in the primary task, it is suggested to compare the articulatory suppression condition with another dual-task condition. This ensures that the cognitive load across the two conditions is comparable and the performance impairment in the primary task is selectively due to linguistic interference rather than a cognitive load disparity between conditions. Then, the authors’ attention shifts to choosing the most appropriate control secondary task. A key aspect considered when selecting a control condition is the cognitive load imposed by the secondary tasks. The control secondary task matches the experimental secondary task in several aspects, depending on the modalities chosen for the articulatory suppression, so that any observed effect on the dependent variable can be attributed to the independent variable itself, rather than to a mismatch in cognitive demands, for example, between the two secondary tasks. Choosing a secondary task in the experimental condition that requires greater cognitive resources than that in the control condition would introduce a confound. Consequently, any differences in primary task performance could be explained by the higher cognitive demand of the experimental secondary task rather than by the intended manipulation. In contrast to the previous case, articulatory suppression is also employed as a control task. This use is particularly common in studies investigating aspects of working memory unrelated to verbal processing, such as visual working memory (Chung et al., 2025; Sun et al., 2021). In such studies, researchers seek to ensure that performance in tasks such as object memorization reflects specifically the cognitive processes underlying visual working memory, while minimizing the potential confounding influence of verbal recoding strategies.

## 4 Discussion and proposal

This mini-review focuses on studies that specifically use articulatory suppression to disrupt phonological access. We examined the different methodologies and aims underlying the implementation of articulatory suppression tasks. First, we observed a substantial methodological heterogeneity, ranging from the articulated stimuli, articulation modality, and rhythm to the presentation format. These variations have direct consequences for the cognitive and sensorimotor processes involved, and for their relative impact on more or less embodied forms of inner speech because, depending on the adopted approach, both the quantity and the quality of sensorimotor processes—being flexible and context-dependent (Barsalou et al., 2008; Kemmerer, 2019)—may vary and underlie different neurophysiological mechanisms. Importantly, sensorimotor simulation processes, as shown in numerous studies, are culturally mediated (Ghandhari et al., 2020; for a full review, see Ibáñez et al., 2023), and the effect of articulatory processes on inner speech will likely vary depending on the relationship between the participant’s native language and the language of the stimuli. In light of this, it appears clear how the quality of articulatory suppression can differently interfere with inner speech, which can be conceived “as a physical process that unfolds over time, leading to an enactive re-creation of auditory percepts via the simulation of articulatory actions” (Nalborczyk et al., 2022; Fini et al., 2022). Depending on the modality of the parameters adopted in the articulatory suppression implementation, and depending on the type of cognitive task aiming to elicit inner speech, different sensorimotor and cognitive processes can be more or less efficiently captured.

For this reason, we suggest that further studies should explore more in depth the neurocognitive implications of the different modalities of articulatory suppression. It would indeed be useful for researchers working in the field to be aware of the cognitive implications of asking: (i) participants to articulate meaningful syllables or words rather than meaningless ones; (ii) to produce syllables composed of open vowels rather than closed ones; (iii) to articulate aloud rather than whisper, or even just move the mouth without articulating (e.g., chewing). We also report that articulatory suppression can be used both as main experimental condition and control condition. In both cases, the use of articulatory suppression is quite heterogeneous and sometimes not sufficiently described. Furthermore, a lack of attention to methodological details characterizes studies where articulatory suppression is employed as control condition. This knowledge would be helpful in adopting more systematic methodological choices that rely on a clearer classification of articulatory suppression forms; this might contribute to further understanding their differential effects on cognitive tasks. Such an approach is essential for refining dual-task paradigms and ensuring more robust experimental designs, thus fostering research on the embodied nature of language and inner speech.
