# Supplementary material for: Variability and methodological choices in articulatory suppression tasks: a review
Source: Front Psychol. 2026 Mar 18;17:1736170. doi: 10.3389/fpsyg.2026.1736170 (PMC13038976; doi:10.3389/fpsyg.2026.1736170)
Supplement: Supplementary file 1 [file Table_1.docx]

**Table 1**

*The table summarizes studies published between 2015 and 2025 (n = 72) that employed articulatory suppression (AS) as the primary experimental manipulation (authors and year are reported in columns 11 and 12). It details key characteristics of the AS procedure, including the AS stimulus (column 1), its modality (column 2), rhythm (column 3), the method used to monitor AS performance accuracy (column 4), and the presentation format (column 5). In addition, the table indicates whether AS was implemented in combination with other secondary tasks and control conditions (column 9), and specifies the particular sub-study or sub-studies in which AS was used. . Finally, it reports the primary task(s), dependent variable(s), and stimulus materials used in each study.*

| **Stimulus of the AS** | **Modality of the AS** | **Rhythm of the AS** | **AS Monitoring** | **Presentation Format of the AS Stimulus** | **AS - AS Control Condition - Other Secondary Task** | **Primary Task** | **Dependent Variable of the Primary Task** | **Stimulus of the Primary Task** | **Language** | **Author(s)** | **Year** |
| --- | --- | --- | --- | --- | --- | --- | --- | --- | --- | --- | --- |
| Seven consonants | Repeating aloud | Unspecified | Unspecified | Written | AS, visual control condition | WMT (visual) | Accuracy | Videos of real-word scenes | Unspecified (likely EN) | Cronin et al. | 2020 |
| 4 syllables "re, ab, mei, la, fi, ex, er, be, kon, de, un"; four letters "K, P, G, L, E, F, O, C, A, B, D, M" | Silently in the head | Unspecified | Unspecified | Written | AS, no AS control condition (E1, E2) | Object naming, Verbalization task | Speech onset latency | Objects (visually), actions (pictures, visually) | DE | Gerwien et al. | 2022 |
| Alphabetic sequence "a,b,c,d,e,f,g" | Repeating aloud | Two letters per second | Unspecified | Written | AS, no AS control condition (E2-E6) | Serial recall | Recall performance, articulation time | Words (long vs. short) | FR | Guitard et al. | 2018 |
| German syllables (‘ar’, ‘en’, ‘ip’, ‘ot’, ‘uk’, ‘be’, ‘to’, ‘su’, ‘li’, ‘ke’) | Repeating aloud | Unspecified | Experimenter | Auditorily | AS, visual tracking task | Discrimination task (auditory and visual) | Sensitivity, bias | Auditorily and visual stimuli | DE | Bratzke et al. | 2016 |
| Letters "A, B, C, D" | Repeating aloud | One letter every 350ms | Experimenter | Written | AS, no control condition (E4) | Serial recall (ear-alternating items) | Recall performance | Spoken digits 1–8 | FR | Hughes et al. | 2016 |
| Letters "a, e, i, o, u, a, e, i, o, u" | Unspecified | One letter per beat | Unspecified | Auditory | AS (E3), anagram task and visual search task (E1, E2) | Recognition task | Recognition performance | Four-letter nouns | JA | Miura and Itoh | 2016 |
| Letters "a,b,c,d" ; Numbers "1,2,3,4" | Unspecified | Unspecified | Unspecified | Unspecified | AS, no control condition (E1a,b; E2a-c) | WMT (non-spatial; spatial) | Reaction times, accuracy | Geometric colored shapes | Unspecified | Ahn et al. | 2017 |
| Non-word "BABATAKA" (E2) | Repeating aloud (E1, E2) | Seven times over 10.5s (E1, E2) | Unspecified | Written | AS (backward counting), finger tapping (E1); AS (E2) | Recall task (cued sentence) | Sentence recall accuracy (E1, E2) | Auditorily presented sentences with homophone vs. non-homophone words | EN | Meltzer et al. | 2016 |
| Number "two" | Unspecified | Six times | Unspecified | Written | AS, low cognitive load motor task, high cognitive load motor task (E1) | Complex span task | Serial recall | Sentences describing actions | Unspecified | Plancher et al. | 2019 |
| Number (33-99). Repeating a new number each trial. | Repeating aloud | Unspecified | Unspecified | Written | AS, no AS control condition (E1-E3) | Change-detection task (item-item binding) | Hits, false alarms, A', sensitivity | Faces | Unspecified (likely EN) | Peterson and Naveh-Benjamin | 2017 |
| Number (unspecified) | Unspecified | Unspecified | Unspecified | Written | AS (E1); AS, backward counting (E2) | Change-detection task | Hits, false alarms, A', sensitivity | Faces | Unspecified (likely EN) | Peterson et al. | 2019 |
| Numbers "3-1-2-4" | Unspecified | As fast and as accurate as possible | Unspecified | Unspecified | AS, spatial tapping | WMT: Letter reading span task, symmetry span task | Accuracy | Digits, letters, grids (visually) | Unspecified (likely RU) | Izmalkova et al. | 2022 |
| Numbers "8, 9, 10" | Whispering | 3 items | Audio-video recording | Unspecified | AS, no AS control condition | Serial recall task | Recall performance | Random permutations of consonants (auditory or visual) | Unspecified (likely EN) | Macken et al. | 2016 |
| Numbers "one, two, three" | Unspecified | Unspecified | Audio-recording | Oral | AS, no AS control condition, gestural interference | Recall task (gestures) | Correct movements | Video sequences of gestures | FR | Gimenes et al. | 2016 |
| Numbers “1, 2, 3" | Repeating aloud | 1 per second (training: repeat at consistent rate) | Unspecified | Unspecified | AS, no control condition | Sentence acceptability judgment | Response time, error rate | Sentences with homophone vs. non-homophone words | Kanji | Morita and Saito | 2019 |
| Numbers (count aloud from 1) | Counting aloud ( | Unspecified | Unspecified | Written | AS, no AS control condition, visual distractor task (E2) | Recognition task (items) | Sensitivity, response bias, serial position, dissimilarity | Sounds | Unspecified | Siedenburg and McAdams | 2017 |
| Numbers (count from 1-7) | Counting aloud | Continuously (each digit half a second or less to say) | Recorded using an MP3 player | Unspecified | AS, irrelevant speech, thinking aloud, silent working | Insights problems | Proportion of problems solved | Pictorial problems | Unspecified (likely EN) | Ball et al. | 2019 |
| Solved math problems (E1, E2) | Repeating aloud (E1); mouthing (E2) | Unspecified | Experimenter | Written | "AS, no AS control condition, attentional refreshing suppression, AS + attentional refreshing suppression (E1); E2: same but mouthed AS | Melody comparison task | Sensitivity (E1, E2) | Nonverbal sounds | Unspecified | Nees et al. | 2017 |
| Solved math problems (E1, E2) | Reading aloud | Fast, consistent | Experimenter | Written | AS, no AS control condition, attentional refreshing suppression, AS + attentional refreshing suppression (E1, E2) | Timbre memory task with three (E1) and two (E2) sound sequences | Sensitivity (E1, E2) | Abstract sounds | Unspecified | Nees and Leong | 2018 |
| Syllable "ba" | Unspecified | 1Hz | Unspecified | Unspecified | AS, no AS control condition (E1-E3) | WMT: Memory and processing tasks | Span | Letter (visually + aurally) | EN; FR | Belletier et al. | 2023 |
| Syllable "ba" | Unspecified | 86 beats per minute | Metronome | Written | AS, no control condition (E2) | Span task | Percentage of correct responses | Series of consonants, ideograms | ZH | Barrouillet et al. | 2015 |
| Syllable "bababa" | Repeating aloud | Continuously | Experimenter | Written | AS, no AS control condition, color labelling | WMT (visual) | Recall performance | Sequence of colors | Unspecified | Forsberg et al. | 2017 |
| Syllable "da" | Unspecified | Every 600 ms | Unspecified | Visual | AS, key tapping (E1) | WMT (auditory) | Accuracy (E1) | Artificial sounds | Unspecified | Soemer and Saito | 2015 |
| Syllable "fi" | "Repeating continuously | E5: 60 beat per minute | Audio-recording | Unspecified | AS, foot tapping (tap to the beat of a metronome (60 bpm); E5) | Contingent capture | Accuracy; reaction time | Color words | Unspecified | Baier and Ansorge | 2019 |
| Syllable "la" | Unspecified | 2 Hz | Unspecified | Unspecified | AS, no AS control condition | Picture naming task | Response time, error rate | Words (visually, auditorily) | EN | Tsuboi et al. | 2020 |
| Syllable "la" (E1); Numbers between 20-99 (E2) | Whispering (E1); unspecified (E2) | Unspecified | Unspecified | Written (E1-E2) | AS (E1); AS, count upwards (E2) | WMT (visual) | Accuracy (E1) | Geometric colored shapes | EN | Atkinson et al. | 2018 |
| Syllable "la" | Repeating aloud | Three times per second | Unspecified | Unspecified | AS, no control condition (E3) | Arabic number comparison task | Reaction times, errors | Number pairs | IT | Gazzellini and Laudanna | 2015 |
| Syllables "ba bi bu" | Repeating continuously | Self-chosen rate | Unspecified | Visual | AS, no control condition (E3) | Serial recall | Recall performance | Chunks | DE | Thalmann et al. | 2019 |
| Syllables "ba-bi-boo" | Repeating aloud | Continuously | Unspecified | Written | AS, no control condition | Span task with immediate and delayed recall | Conditionalized, free, and serial scoring | Concrete words | FR | Abadie and Camos | 2018 |
| Syllables "ba-bi-bou" | Unspecified | One syllable every 500ms | Metronome | Unspecified | AS, no control condition (E2) | Brown–Peterson task; parity judgment task | Correct responses, recall performance | Letters | Unspecified (likely FR) | Belletier and Camos | 2018 |
| Syllables "ba, ba, ba" | Unspecified | Unspecified | Unspecified | Unspecified | AS, no control condition (E4) | Event file binding task | Response time | Square (perception task), white outline square with “R” or “G” (imagery task) | Unspecified (likely EN) | Cochrane and Milliken | 2019 |
| Syllables "ba, di, boo" | Unspecified | One syllable every 750ms | Unspecified | Unspecified | AS, no control condition (E2a-b) | Working memory task (auditory, visual) | Correct recall | Lines of dots | Unspecified (likely FR) | Uittenhove et al. | 2019 |
| Syllables "bababa" | Repeating aloud | Continuously | Audio-recording | Written | "AS, color labelling (E1); AS, color labeling, position labeling, preference rating + AS (E2); AS, color labeling, remember label (E3); no AS control condition, AS (label group; E4); no AS control condition, AS (silent group; E4) | WMT (visual) | Probability of WM storage, probability of continuous representation, imprecision of continuous memory, probability of categorical guessing; category selectivity, category imprecision | Sequence of colors | Unspecified | Souza and Skora | 2017 |
| Syllables "babibu" | Repeating aloud | Unspecified | Unspecified | Written | AS, articulatory rehearsal (E3) | Delayed-recognition test (elaboration vs. no elaboration) | Reaction times, hit rates recall | List of words (differing in concreteness and imageability) | DE | Thalmann et al. | 2019 |
| Syllables "babibu" | Unspecified | Unspecified | Unspecified | Visual | AS, no control condition (E3) | Serial recall | Recall performance | Series of letters | Unspecified | Vergauwe | 2018 |
| Syllables "badibu" | Unspecified | Every 750 ms | Unspecified | Unspecified | AS, no control condition | Serial recall task | Recall performance | Series of letters | Unspecified | De Schrijver and Barrouillet | 2017 |
| Syllables "bibibibi" | Repeating aloud | Unspecified | Audio-recording every eighteenth trial | Unspecified | AS, no control condition | Serial recall task | Recall performance, serial recall, temporal distinctiveness | Lists of colors | Unspecified | Peteranderl and Oberauer | 2018 |
| Syllables "bla-bli-blu" | Repeating aloud | Unspecified | Unspecified | Written | AS, no control condition | Comparative visual search task | Response times, error rate | Colored circles, animals silhouettes | Unspecified | Hardiess and Mallot | 2015 |
| Syllables "blah blah" | Repeating aloud | Unspecified | Experimenter | Written | AS, no control condition | WMT (auditory, tactile, visual-spatial) | WM performance; sensitivity | Memory arrays of rectangles, auditory cues, vibrotactile cues | Unspecified | Curtis et al. | 2019 |
| Syllables "la la la" | Repeating aloud | Quickly | Unspecified | Unspecified | AS, no control condition | Digit span test | Span measure | Random sequence of numbers (1 to 9) in different modalities (auditory; visual; audiovisual) | IT | Talamini et al. | 2016 |
| Syllables “babibu” | Repeating aloud | 6 times | Controlled by visually displaying which words participants uttered | Written on screen | Articulatory suppression (fast condition, slow AS, slow rehearsal; E3) | Memory task | Correct recall word, position | Concrete and abstract words | DE | Souza and Oberauer | 2018 |
| Syllables “ta”, “da” | Repeating aloud | Unspecified | Unspecified | Unspecified | AS, no AS control condition | Visual change-detection task | Detection performance | Abstract patterns | Unspecified | Sense et al. | 2017 |
| Syllables (“ma”, “na”, “la”, “sa”, “mo”, “no”, “lo”, “so”). Participants were allowed to change the syllable, among the eight propositions, after each trial. | Repeating aloud | At comfortable pace, without distinct breaks | Audio-recording | Visual (fixation cross) | AS, no control condition | Lexical decision task, prime visibility task | Reaction times, errors | Words, non-words | FR | Sun and Peperkamp | 2016 |
| Three digits (E1); three shapes (E2) | Repeating aloud (E1, E2) | Unspecified | Unspecified | Written (E1, E2) | AS, no control condition (E1); AS (E2) | Scene encoding | Recognition performance (E1, E2) | Scenes | EN | Rehrig et al. | 2020 |
| Two digits between 1-9 | Repeating aloud | Unspecified | Unspecified | Written | AS, no control condition (E1-E3) | Change-detection task | Perimetric complexity, performance | EN letters (E1); letters from Braille, Hebrew, Arabic, ZH (E2); Brussels Artificial Character Set (E3) | EN | Ngiam et al. | 2019 |
| Unspecified | Unspecified | Unspecified | Unspecified | Unspecified | AS, no control condition | Simultaneous interpreting (free recall, comprehension questions, cloze task) | Accuracy, comprehension | Speech | EN | Díaz-Galaz and Torres | 2019 |
| Word "bee"; Alphabetic sequence ("a" to "g") | Mouthing | Twice per second | Video camera + experimenter | Unspecified | AS, no AS control condition (E2) | Recall task (spatial and verbal memory) | Proportion of correct recall | Sequence of consonants; sequence of dots | FR | Guitard and Saint-Aubin | 2015 |
| Word "blah blah" | Unspecified | Unspecified | Unspecified | Auditory | AS, spatial task (E1, E2) | Consistency paradigm | Reading time, errors | Short narratives | Unspecified (likely ES) | Irrazabal and Burin | 2016 |
| Word "California" | Repeating aloud | Unspecified | Experimenter | Unspecified | AS, no control condition | Nonverbal counting task | Performance | Numerals, dot arrays | EN | Au et al. | 2018 |
| Word "Coca-cola" | Repeating (unspecified; | Unspecified | Unspecified | Unspecified | AS, no control condition (E1-E3) | Change-detection task | Response times, hit rates | Textures, colors, shapes | Unspecified (likely EN) | Udale et al. | 2017 |
| Word "Cola" | Repeating aloud | Unspecified | Unspecified | Unspecified | AS, tone discrimination task (single task) | Memory retro-cue paradigm | WM accuracy, response time | Colored circles (visually) | Unspecified | Lin et al. | 2021 |
| Word "mathématiques" | Unspecified | Continuously, three utterances every 2s | Unspecified | Unspecified | AS, spatial suppression (tapping at a rhythm of approximately 2 taps per second; E1) | Visuo-spatial and verbal-control versions of the Brooks matrix task | Number of correct recall | Matrices | FR | Poirier et al. | 2019 |
| Word "Monday" | Unspecified | Unspecified | Unspecified | Unspecified | AS, no control condition (E3) | WMT | Proportion of correct recall | Lists of letters | Unspecified (likely EN) | Bayliss et al. | 2015 |
| Word "oui" (E2); Syllables "ba bi boo" (E3, E4) | Repeating aloud (E2); unspecified (E3, E4) | Three digits per second (E2); Continuously (E3, E4) | Unspecified | Auditory (E2); Written (E3, E4) | AS, no AS control condition, location judgement task, AS + location judgment task (E2); AS + parity judgment task (E3, E4) | Span task (E1,E2); Brown-Peterson tasks (E3) | Recall performance (E2); recall performance, response times (E3, E4) | Words, non-words | FR | Camos et al. | 2019 |
| Word "racket" | Repeating aloud | Continuously | Experimenter | Unspecified | AS, no control condition | Homophone and rhyme judgment task | Proportion of correct detection, number of correct recall, false alarms, reaction times | Words (homophone vs. rhyme) | EN | Norris et al., | 2017 |
| Word "the" | Repeating aloud | Twice per second | Monitored by the experimenter | Unspecified | AS (E1, E2a, E2b), No AS condition, E3 auditory monitoring control task | Memory for events | Performance score, completion time | Constructing object through videos (visually) | EN | Banks & Connell | 2024 |
| Word "the" | Repeating aloud | Both secondary tasks at approximately the same rhythmic rate | Unspecified | Orally + showed | AS, no AS control condition (E1), foot tapping (E2) | Memory for sequences of objects | Accuracy, response time | Images of natural and artifacts objects | EN | Dymarska et al. | 2022 |
| Word "the" (E1-E3) | Repeating aloud (E1-E3) | Unspecified | Research assistants | AS green cross presented on the screen + forget cue; late AS green cross (E1); AS green cross presented on the screen + forget cue (E2) | AS, no AS control condition, late AS (E1); AS, no AS control condition (E2); AS (E3) | Directed forgetting task | False alarm to negative probes, accuracy to positive probes, accuracy in LTM recognition test (E1, E2, E3) | Words | EN | Festini and Reuter-Lorenz | 2017 |
| Word "the" | Repeating aloud | Twice per second | Unspecified | Written | AS, no control condition (E1-E5) | Serial recall and recognition task (E1, E2: Chunks recognition; E3: serial recall; E4: chunks recognition, E5: serial recall) | Recognition accuracy (E1, E2); recall performance (E3); recognition accuracy (E4); recall performance (E5) | Lists of words | Unspecified (likely EN) | Norris et al. | 2020 |
| Word "the" | Repeating aloud | Twice per second | Experimenter | Auditory | AS, no AS control condition (E1, E2) | Recall task (auditory sequences) | Proportion of correct recall | Sequences of spoken digits 1–9; irrelevant sounds | EN | Hanley and Bourgaize | 2018 |
| Word "the" | Unspecified | Two words per second, 24 times | Unspecified | Written | AS, no AS control condition (E1-E2) | Serial recall task | Recall performance (proportion of correct responses, E1, E2) | Words (visually similar vs. dissimilar; E1) | EN | Guitard and Cowan | 2020 |
| Word "the" | Audibly at any volume they wished | 3 times per second(metronome) | Metronome | Auditory, onset metronome | AS, no AS control condition, maintenance (E1) | Change-detection task | Performance, rate of forgetting | Arrays of letters | EN | Ricker et al., | 2020 |
| Word "the" (E1) | Repeating aloud (E1) | Unspecified | Unspecified | Oral (E1); Visually (E2) | AS, no control condition (E1, E2) | Recognition task (objects memory) | Accuracy, response time (E1); accuracy, reaction times (E2) | Sequences of real-world objects | EN | Dymarska et al. | 2019 |
| Word "the" (E1b-c, E2a-b) | Unspecified | Twice per second (E1b); 1 per second (E1c, 2A); 1.5 responses per second (E2B) | Unspecified | Unspecified | Finger tapping (tap at a rate of 2 responses per second; E1a); articulatory suppression (E1b-c); Finger tapping + articulatory suppression only during ISIs (tap at a rate of 2 responses per second; E2a); Finger tapping + articulatory suppression (tap at rate of 2 responses per second; E2b). | Recognition task (nonwords); Recognition task (synonyms; E1a-c) | Proportion of correct responses (E1b-c), mean recognition rate | Nonwords, synonyms (E1a-c); nonwords, synonyms (E2a-b) | Kanji and Hiragana (for synonym recognition task) | Nishiyama | 2018 |
| Word "the" | Unspecified | Two words per second | Unspecified | Unspecified | AS (E1b), no AS control condition (E1a); | Probed serial-recall | Accuracy | Chinese characters | ZH | Lin et al. | 2015 |
| Word "the" | Subvocal articulation | Unspecified | Unspecified | Unspecified | AS, no AS control condition, finger tapping (E2, E3) | Short term memory task | Reaction times, accuracy | Audio recordings (E2) | Unspecified (likely EN) | Klyn et al. | 2016 |
| Word "the" (E3); Numbers "1,2,3,4" (E4) | Repeat (unspecified) | Unspecified | Audio recording | Unspecified | AS, no AS control condition (E3-E4) | Serial recall task | Recall performance (accuracy, serial position curves, probability of first recall; E3, E4) | Rectangles (E3); words, circles (E4) | Unspecified (likely EN) | Cortis et al. | 2015 |
| Word "tick" (E1, E2) | Unspecified | 1,67 Hz (100 beats per minute) (E1, E2) | Metronome | Unspecified | AS, foot tapping (E1); AS (E2) | WMT: Visuospatial encoding task | % error, Reaction time | Figures of objects (visually) | EN | van’t Wout and Jarrold | 2022 |
| Word "Vanderbilt" | Repeating aloud | Three clicks (0.66s, 1.33s, and 2s) every two seconds (metronome) | Unspecified | Auditory | AS (E3); spatial orientation task (E4) | Partial-report task | Numbers of targets and distractors reported | Letters | Unspecified (likely EN) | Lindsey et al. | 2017 |
| Word not related to the stimulus (unspecified) | Unspecified | Unspecified | Unspecified | Unspecified | Digit span, listening span, digit span with articulatory suppression, listening span with articulatory suppression | Simultaneous interpreting | Fluency, delay, accuracy, quality of the simultaneous interpreting, simultaneous interpreting global score | Speech | EN | Injoque-Ricle et al. | 2015 |
| Words "bla bla" | Unspecified | Unspecified | Research assistant | Written | AS, spatial tapping | Read and executed instructions to assemble a LEGO | Study times; mean error rates | Instructions to assemble lego object | Unspecified | Irrazabal et al. | 2016 |

**References**

Abadie, M., and Camos, V., 2018. Attentional refreshing moderates the word frequency effect in immediate and delayed recall tasks. Annals of the New York Academy of Sciences, 1424(1), 127–136. <https://doi.org/10.1111/nyas.13847>

Ahn, J., Patel, T. N., Buetti, S., and Lleras, A., 2017. Exploring the contributions of spatial and non-spatial working memory to priming of pop-out. Attention, Perception, & Psychophysics, 79(4), 1012–1026. https://doi.org/10.3758/s13414-017-1285-x

Atkinson, A. L., Berry, E. D., Waterman, A. H., Baddeley, A. D., Hitch, G. J., and Allen, R. J., 2018. Are there multiple ways to direct attention in working memory? Annals of the New York Academy of Sciences, 1424(1), 115–126. <https://doi.org/10.1111/nyas.13634>

Au, J., Jaeggi, S. M., and Buschkuehl, M., 2018. Effects of non-symbolic arithmetic training on symbolic arithmetic and the approximate number system. Acta Psychologica, 185, 1–12. <https://doi.org/10.1016/j.actpsy.2018.01.005>

Baier, D., and Ansorge, U., 2019. Investigating the role of verbal templates in contingent capture by color. Attention, Perception, & Psychophysics, 81(6), 1846–1879. <https://doi.org/10.3758/s13414-019-01701-y>

Ball, L. J., Marsh, J. E., Litchfield, D., Cook, R. L., and Booth, N., 2019. When distraction helps: Evidence that concurrent articulation and irrelevant speech can facilitate insight problem solving. In Insight and creativity in problem solving (pp. 76–96). Routledge.

Banks, B. and Connell, L., 2024. Access to inner language enhances memory for events. Journal of Experimental Psychology: Learning, Memory, and Cognition.

Barrett, H. C. (2012). A hierarchical model of the evolution of human brain specializations. Proceedings of the national Academy of Sciences, 109(supplement_1), 10733-10740.

Barrouillet, P., Corbin, L., Dagry, I., and Camos, V., 2015. An empirical test of the independence between declarative and procedural working memory in Oberauer’s (2009) theory. Psychonomic Bulletin & Review, 22(4), 1035–1040. <https://doi.org/10.3758/s13423-014-0787-y>

Bayliss, D. M., Bogdanovs, J., and Jarrold, C., 2015. Consolidating working memory: Distinguishing the effects of consolidation, rehearsal and attentional refreshing in a working memory span task. Journal of Memory and Language, 81, 34–50. <https://doi.org/10.1016/j.jml.2014.12.004>

Belletier, C., and Camos, V., 2018. Does the experimenter presence affect working memory? Annals of the New York Academy of Sciences, 1424(1), 212–220. <https://doi.org/10.1111/nyas.13627>

Belletier, C., Doherty, J. M., Graham, A. J., Rhodes, S., Cowan, N., Naveh-Benjamin, M., Barrouillet, P., Camos, V., and Logie, R. H., 2023. Strategic adaptation to dual-task in verbal working memory: Potential routes for theory integration. Journal of Experimental Psychology: Learning, Memory, and Cognition, 49(1), 51–77. <https://doi.org/10.1037/xlm0001106>

Bratzke, D., Quinn, K. R., Ulrich, R., and Bausenhart, K. M., 2016. Representations of temporal information in short-term memory: Are they modality-specific? Acta Psychologica, 170, 163–167. https://doi.org/[10.1016/j.actpsy.2016.08.002](https://doi.org/10.1016/j.actpsy.2016.08.002)

Camos, V., Mora, G., Oftinger, A.-L., Mariz Elsig, S., Schneider, P., and Vergauwe, E., 2019. Does semantic long-term memory impact refreshing in verbal working memory? Journal of Experimental Psychology: Learning, Memory, and Cognition, 45(9), 1664–1682. https://doi.org/[10.1037/xlm0000657](https://doi.org/10.1037/xlm0000657)

Cochrane, B. A., and Milliken, B., 2019. Imagined event files: An interplay between imagined and perceived objects. Psychonomic Bulletin & Review, 26(2), 538–544. <https://doi.org/10.3758/s13423-019-01572-2>

Cortis, C., Dent, K., Kennett, S., and Ward, G., 2015. First things first: Similar list length and output order effects for verbal and nonverbal stimuli. Journal of Experimental Psychology: Learning, Memory, and Cognition, 41(4), 1179–1214. https://doi.org/[10.1037/xlm0000086](https://doi.org/10.1037/xlm0000086)

Cronin, D. A., Peacock, C. E., and Henderson, J. M., 2020. Visual and verbal working memory loads interfere with scene-viewing. Attention, Perception, & Psychophysics, 82(5), 2814–2820. <https://doi.org/10.3758/s13414-020-02076-1>

Curtis, A. F., Turner, G. R., Park, N. W., and Murtha, S. J., 2019. Improving visual spatial working memory in younger and older adults: Effects of cross-modal cues. Aging, Neuropsychology, and Cognition, 26(1), 24–43. <https://doi.org/10.1080/13825585.2017.1397096>

De Schrijver, S., and Barrouillet, P., 2017. Consolidation and restoration of memory traces in working memory. Psychonomic Bulletin & Review, 24(6), 1651–1657. <https://doi.org/10.3758/s13423-017-1226-7>

Díaz-Galaz, S., and Torres, A., 2019. Comprehension in interpreting and translation: Testing the phonological interference hypothesis. Perspectives, 27(4), 622–638. <https://doi.org/10.1080/0907676X.2019.1569699>

Dymarska, A., Connell, L., and Banks, B., 2019. Working memory for object concepts relies on both linguistic and simulation information. In Proceedings of the Annual Meeting of the Cognitive Science Society (Vol. 41). <https://escholarship.org/uc/item/99q0n9dm>

Dymarska, A., Connell, L., and Banks, B., 2022. Linguistic bootstrapping allows more real-world object concepts to be held in mind. Collabra: Psychology, 8(1), Article 40171. <https://doi.org/10.1525/collabra.40171>

Festini, S. B., and Reuter-Lorenz, P. A., 2017. Rehearsal of to-be-remembered items is unnecessary to perform directed forgetting within working memory: Support for an active control mechanism. Journal of Experimental Psychology: Learning, Memory, and Cognition, 43(1), 94–108. <https://doi.org/10.1037/xlm0000308>

Forsberg, A., Johnson, W., and Logie, R. H., 2020. Cognitive aging and verbal labeling in continuous visual memory. Memory & Cognition, 48(7), 1196–1213. <https://doi.org/10.3758/s13421-020-01043-3>

Gazzellini, S., and Laudanna, A., 2015. Digit repetition effect in two-digit number comparison. Zeitschrift für Psychologie. https://doi.org/10.1027/2151-2604/a000043

Gerwien, J., von Stutterheim, C., and Rummel, J., 2022. What is the interference in “verbal interference”? Acta Psychologica, 230, Article 103774. <https://doi.org/10.1016/j.actpsy.2022.103774>.

Gimenes, G., Pennequin, V., and Mercer, T., 2016. What is the best strategy for retaining gestures in working memory? Memory, 24(6), 757–765. https://doi.org/10.1080/09658211.2015.1049544

Guitard, D., and Cowan, N., 2020. Do we use visual codes when information is not presented visually? Memory & Cognition, 48(8), 1522–1536. https://doi.org/10.3758/s13421-020-01054-0

Guitard, D., and Saint-Aubin, J., 2015. A replication of “Functional equivalence of verbal and spatial information in serial short-term memory (1995; Experiments 2 and 3)”. Memory & Cognition, 43(4), 1008–1018. <https://doi.org/10.3758/s13421-015-0554-y>

Guitard, D., Saint-Aubin, J., Tehan, G., and Neath, I., 2018. Does neighborhood size really cause the word length effect? Memory & Cognition, 46(2), 244–260. <https://doi.org/10.3758/s13421-017-0761-9>

Hanley, J. R., and Bourgaize, J., 2018. Similarities between the irrelevant sound effect and the suffix effect. Memory & Cognition, 46(6), 841–848. https://doi.org/10.3758/s13421-018-0806-8

Hardiess, G., and Mallot, H. A., 2015. Allocation of cognitive resources in comparative visual search–Individual and task dependent effects. Vision Research, 113, 71–77. https://doi.org/10.1016/j.visres.2015.05.017

Holt, J. L., and Delvenne, J. F., 2015. A bilateral advantage for maintaining objects in visual short term memory. Acta Psychologica, 154, 54–61. https://doi.org/10.1016/j.actpsy.2014.11.007

Hughes, R. W., Chamberland, C., Tremblay, S., and Jones, D. M., 2016. Perceptual-motor determinants of auditory-verbal serial short-term memory. Journal of Memory and Language, 90, 126–146. https://doi.org/10.1016/j.jml.2016.04.006

Injoque-Ricle, I., Barreyro, J. P., Formoso, J., and Jaichenco, V. I., 2015. Expertise, working memory and articulatory suppression effect: Their relation with simultaneous interpreting performance. Advances in Cognitive Psychology, 11(2), 56–63. <https://doi.org/10.5709/acp-0171-1>

Irrazabal, N., and Burin, D., 2016. Spatial inferences in narrative comprehension: The role of verbal and spatial working memory. The Spanish Journal of Psychology, 19, E11. [https://doi.org/10.1017/sjp.2016.11](https://psycnet.apa.org/doi/10.1017/sjp.2016.11)

Irrazabal, N., Saux, G., and Burin, D., 2016. Procedural multimedia presentations: The effects of working memory and task complexity on instruction time and assembly accuracy. Applied Cognitive Psychology, 30(6), 1052–1060. https://doi.org/10.1002/acp.3299

Izmalkova, A., Barmin, A., Velichkovsky, B. B., Prutko, G., and Chistyakov, I., 2022. Cognitive resources in working memory: Domain-specific or general? Behavioral Sciences, 12(11), Article 459. <https://doi.org/10.3390/bs12110459>

Klyn, N. A., Will, U., Cheong, Y. J., and Allen, E. T., 2016. Differential short-term memorisation for vocal and instrumental rhythms. Memory, 24(6), 766–791. https://doi.org/10.1080/09658211.2015.1050400

Lin, Y. C., Chen, H. Y., Lai, Y. C., and Tzeng, O. J. L., 2015. Phonological similarity and orthographic similarity affect probed serial recall of Chinese characters. Memory & Cognition, 43(4), 538–554. https://doi.org/10.3758/s13421-014-0495-x

Lin, Y. T., Kong, G., and Fougnie, D., 2021. Object-based selection in visual working memory. Psychonomic Bulletin & Review, 28(6), 1961–1971. https://doi.org/10.3758/s13423-021-01971-4

Lindsey, D. R. B., Bundesen, C., Kyllingsbæk, S., and Jensen, J., 2017. Out with the old? The role of selective attention in retaining targets in partial report. Attention, Perception, & Psychophysics, 79(1), 117–137. https://doi.org/10.3758/s13414-016-1214-4

Macken, B., Taylor, J. C., Kozlov, M. D., Hughes, R. W., and Jones, D. M., 2016. Memory as embodiment: The case of modality and serial short-term memory. Cognition, 155, 113–124. <https://doi.org/10.1016/j.cognition.2016.06.013>

Meltzer, J. A., Rose, N. S., Deschamps, T., Osthfeld-Steinberg, S., and Binns, M. A., 2016. Semantic and phonological contributions to short-term repetition and long-term cued sentence recall. Memory & Cognition, 44(2), 307–329. https://doi.org/10.3758/s13421-015-0554-y

Miura, H., and Itoh, Y., 2016. The effect of the feeling of resolution and recognition performance on the revelation effect. Consciousness and Cognition, 45, 100–108. <https://doi.org/10.1016/j.concog.2016.08.002>

Morita, A., and Saito, S., 2019. Homophone advantage in sentence acceptability judgment: An experiment with Japanese Kanji words and articulatory suppression technique. Journal of Psycholinguistic Research, 48(4), 501–518. https://doi.org/10.1007/s10936-018-9615-2

Nees, M. A., and Leong, P., 2018. Articulatory suppression impairs working memory for ostensibly unvocalizable abstract sounds. Auditory Perception & Cognition, 1(1–2), 131–147. <https://doi.org/10.1080/25742442.2018.1534195>

Nees, M. A., Corrini, E., Leong, P., and Harris, J., 2017. Maintenance of memory for melodies: Articulation or attentional refreshing? Psychonomic Bulletin & Review, 24(6), 1964–1970. <https://doi.org/10.3758/s13423-017-1269-9>

Ngiam, W. X. Q., Khaw, K. L. C., Holcombe, A. O., and Goodbourn, P. T., 2019. Visual working memory for letters varies with familiarity but not complexity. Journal of Experimental Psychology: Learning, Memory, and Cognition, 45(10), 1761–1775. <https://doi.org/10.1037/xlm0000682>

Nishiyama, R., 2018. Separability of active semantic and phonological maintenance in verbal working memory. PLoS ONE, 13(3), Article e0193808. <https://doi.org/10.1371/journal.pone.0193808>

Norris, D., Butterfield, S., Hall, J., and Page, M. P. A., 2018. Phonological recoding under articulatory suppression. Memory & Cognition, 46(2), 173–180. <https://doi.org/10.3758/s13421-017-0754-8>

Norris, D., Kalm, K., and Hall, J., 2020. Chunking and redintegration in verbal short-term memory. Journal of Experimental Psychology: Learning, Memory, and Cognition, 46(5), 872–893. https://doi.org/10.1037/xlm0000762

Oberauer, K., 2022. When does working memory get better with longer time? Journal of Experimental Psychology: Learning, Memory, and Cognition, 48(12), 1754–1774. https://doi.org/[10.1037/xlm0001199](https://doi.org/10.1037/xlm0001199)

Peteranderl, S., and Oberauer, K., 2018. Serial recall of colors: Two models of memory for serial order applied to continuous visual stimuli. Memory & Cognition, 46(1), 1–16. <https://doi.org/10.3758/s13421-017-0741-0>

Peterson, D. J., and Naveh-Benjamin, M., 2017. The role of attention in item-item binding in visual working memory. Journal of Experimental Psychology: Learning, Memory, and Cognition, 43(9), 1403. https://doi.org/10.1037/xlm0000386.supp

Peterson, D. J., Decker, R., and Naveh-Benjamin, M., 2019. Further studies on the role of attention and stimulus repetition in item–item binding processes in visual working memory. Journal of Experimental Psychology: Learning, Memory, and Cognition, 45(1), 56–70. https://doi.org/10.1037/xlm0000577

Plancher, G., Mazeres, F., and Vallet, G. T., 2019. When motion improves working memory. Memory, 27(3), 410–416. https://doi.org/10.1080/09658211.2018.1510012

Poirier, M., Yearsley, J. M., Saint-Aubin, J., and Guérard, K., 2019. Dissociating visuo-spatial and verbal working memory: It’s all in the features. Memory & Cognition, 47(4), 603–618. <https://doi.org/10.3758/s13421-018-0882-9>

Rehrig, G., Hayes, T. R., Henderson, J. M., and Reeder, R. R., 2020. When scenes speak louder than words: Verbal encoding does not mediate the relationship between scene meaning and visual attention. Memory & Cognition, 48(7), 1181–1195. <https://doi.org/10.3758/s13421-020-01050-4>

Ricker, T. J., Sandry, J., Vergauwe, E., and Cowan, N., 2020. Do familiar memory items decay? Journal of Experimental Psychology: Learning, Memory, and Cognition, 46(1), 60–76. https://doi.org/10.1037/xlm0000719

Sense, F., Morey, C. C., Prince, M., Frankish, C., and van der Maas, H. L. J., 2017. Opportunity for verbalization does not improve visual change detection performance: A state-trace analysis. Behavior Research Methods, 49(3), 853–862. <https://doi.org/10.3758/s13428-016-0741-1>

Siedenburg, K., and McAdams, S., 2017. The role of long-term familiarity and attentional maintenance in short-term memory for timbre. Memory, 25(4), 550–564. <https://doi.org/10.1080/09658211.2016.1197945>

Soemer, A., and Saito, S., 2015. Maintenance of auditory-nonverbal information in working memory. Psychonomic Bulletin & Review, 22(6), 1777–1783. https://doi.org/10.3758/s13423-015-0854-z

Souza, A. S., and Oberauer, K., 2018. Does articulatory rehearsal help immediate serial recall? Cognitive Psychology, 107, 1–21. <https://doi.org/10.1016/j.cogpsych.2018.09.002>

Souza, A. S., and Skóra, Z., 2017. The interplay of language and visual perception in working memory. Cognition, 166, 277–297. <https://doi.org/10.1016/j.cognition.2017.05.038>

Sun, Y., and Peperkamp, S., 2016. The role of speech production in phonological decoding during visual word recognition: Evidence from phonotactic repair. Language, Cognition and Neuroscience, 31(3), 391–403. https://doi.org/10.1080/23273798.2015.1100316

Talamini, F., Carretti, B., and Grassi, M., 2016. The working memory of musicians and nonmusicians. Music Perception, 34(2), 183–191. <https://doi.org/10.1525/mp.2016.34.2.183>

Thalmann, M., Souza, A. S., and Oberauer, K., 2019. How does chunking help working memory? Journal of Experimental Psychology: Learning, Memory, and Cognition, 45(1), 37. <https://doi.org/10.5167/uzh-151291>

Thalmann, M., Souza, A. S., and Oberauer, K., 2019. Revisiting the attentional demands of rehearsal in working-memory tasks. Journal of Memory and Language, 105, 1–18. https://doi.org/10.1016/j.jml.2018.10.005

Tsuboi, N., Francis, W. S., and Jameson, J. T., 2020. How word comprehension exposures facilitate later spoken production: Implications for lexical processing and repetition priming. Memory, 29(1), 39–58. <https://doi.org/10.1080/09658211.2020.1845740>

Udale, R., Farrell, S., and Kent, C., 2017. No evidence for binding of items to task-irrelevant backgrounds in visual working memory. Memory & Cognition, 45(7), 1144–1159. https://doi.org/10.3758/s13421-017-0727-y

Uittenhove, K., Chaabi, L., Camos, V., and Barrouillet, P., 2019. Is working memory storage intrinsically domain-specific? Journal of Experimental Psychology: General, 148(11), 2027–2057. https://doi.org/10.1037/xge0000566
