# Supplementary material for: Variability and methodological choices in articulatory suppression tasks: a review
Source: Front Psychol. 2026 Mar 18;17:1736170. doi: 10.3389/fpsyg.2026.1736170 (PMC13038976; doi:10.3389/fpsyg.2026.1736170)
Supplement: Supplementary file 2 [file Table_2.docx]

**Table 2**

*The table summarizes studies published between 2015 and 2025 (n = 77) that employed articulatory suppression (AS) as control condition rather than as the primary experimental manipulation (authors and year are reported in columns 11 and 12). It details key characteristics of the AS procedure, including the AS stimulus (column 1), its modality (column 2), rhythm (column 3), the method used to monitor AS performance accuracy (column 4), and the presentation format (column 5). In addition, the table indicates whether AS was implemented in combination with other secondary tasks and control conditions (column 9), and specifies the particular sub-study or sub-studies in which AS was used.. Finally, it reports the primary task(s), dependent variable(s), and stimulus materials used in each study.*

| **Stimulus of the AS** | **Modality of the AS** | **Rhythm of the AS** | **AS Monitoring** | **Presentation Format of the AS Stimulus** | **AS - AS Control Condition - Other Secondary Task** | **Primary Task** | **Dependent Variable of the Primary Task** | **Stimulus of the Primary Task** | **Language** | **Author(s)** | **Year** |
| --- | --- | --- | --- | --- | --- | --- | --- | --- | --- | --- | --- |
| Four digits | Reading aloud | Unspecified | Unspecified | Written | AS, no control condition (E1, E2) | WMT (visual) | Task performance | Arrays colored squares | Unspecified | Hakim et al. | 2020 |
| Four digits 1-9 | Repeating aloud | Three-four items per second | Experimenter + video recording | Written | AS, no control condition (E1) | WMT (visual) | Accuracy | Colored circles, Landolt-squares | Unspecified | Pan and Zhang | 2020 |
| Four digits 1-9 | Repeating aloud | 3-4 items per second | Unspecified | Written | AS, no control condition (E1) | WMT (visual) | Accuracy; response times | Geometric colored shapes | Unspecified | Pan et al. | 2019 |
| Four letters | Repeating aloud | Unspecified | Unspecified | Unspecified | AS, no control condition (E1-E2) | Visual search task | Reaction times, errors | EN letters | Unspecified (likely EN) | Dube et al. | 2016 |
| Four numbers | Unspecified | Unspecified | Unspecified | Unspecified | AS, no control condition (E3) | WMT (visual, color memory task) | Accuracy (object shape - location) | Colored shapes (visually) | Unspecified (likely EN) | Chung et al. | 2024 |
| Four numbers 1-9 | Repeating aloud | Two repetitions per second | Unspecified | Unspecified | AS, no AS control condition (E1-E4) | WMT (visual) | Accuracy, reaction times, guidance effects | Shapes (E1); colored shapes | Unspecified | Fan et al. | 2019 |
| Korean letters “GaNaDaRa” | Unspecified | Unspecified | Unspecified | Unspecified | AS, no control condition (E2a) | Implicit WMT; visual search task | Response time | Black squares | KO | Ji et al. | 2017 |
| Letters "A-B-C-D" | Repeating aloud | Continuously | Unspecified | Unspecified | AS, no control condition (E1a-b, E2) | Object memory task | Recall performance | Photographs of objects, videos of actions | Unspecified | Lagacé and Guérard | 2015 |
| Letters "A,B,C,D" or numbers "1,2,3,4" | Repeating aloud | Three or four digits/letters per second | Unspecified | Unspecified | AS, no control condition (E1a-b, E2, E3, E4) | WMT (visual) | Change-detection accuracy, response times, sensitivity | Color items in the memory array | Unspecified (likely EN) | Matsukura and Vecera | 2015 |
| Letters "abcd" or "wxyz", numbers "1234" or "6789" | Repeating aloud | Unspecified | Experimenter | Unspecified | AS, no control condition (E6) | Visual search task | Working memory capacity | Photos of real-world objects | EN | Drew et al. | 2016 |
| Number 33-99 (random) | Continuously repeating (no further specified) | Unspecified | Unspecified | Written | AS, backward counting (E1, E2) | WMT | Proportion of correct responses | Black and white line drawings | Unspecified (likely EN) | Sun et al. | 2025 |
| Numbers "1,2,3,4" | Unspecified | Unspecified | Unspecified | Unspecified | AS, no control condition (E1-E3) | Spatial working memory task | Recall performance | Colored shapes | Unspecified | Allen et al. | 2015 |
| Numbers "1,2,3,4" | Repeating aloud | Three digits per second | Experimenter + metronome | Unspecified | AS, no control condition (E2) | Serial recall | Accuracy, transposition gradients, latency serial position curves | Faces | Unspecified | Hurlstone and Hitch | 2018 |
| Numbers "1,2,3" and "2,3,4" | Unspecified | Unspecified | Unspecified | Unspecified | AS, no control condition (E2) | Information binding (shape recognition task) | Percentage of correct responses | Colored geometric shapes | Unspecified | Ferreira and Galera | 2016 |
| Numbers "1,2,3" or "2,3,4" | Repeating aloud | Unspecified | Unspecified | Written | AS, no control condition (E1, E2) | Visual memory and mental imagery tasks | Sensitivity, response times | Sequences of letters (auditorily and visually presented) | Unspecified | Valenti and Galera | 2020 |
| Numbers "123" | Unspecified | Steady rate | Unspecified | Written | AS, backward counting (E1-E7) | WMT (visual) | Proportion of correct recall | Shapes and colors | Unspecified (likely EN) | Allen et al. | 2017 |
| Numbers "123" or "456" or "789" | Repeating aloud | Two numbers per second | Unspecified | Written | AS, no control condition (E3) | Change detection task (visual + tactile) | Recognition performance, accuracy | Attneave shapes | Unspecified | Blalock | 2015 |
| Numbers "1234" | Repeating aloud | Unspecified | Unspecified | Written | AS, no control condition (E1-E2) | Recall task (serial) | Proportion of correct recall | Words with different length | EN | Guitard et al. | 2018 |
| Numbers "two" and "ten" | Repeating aloud | Two words per second | Unspecified | Unspecified | AS, no control condition | Change-detection task (visual) | WM capacity | Colored squares | Unspecified (likely EN) | Morey | 2019 |
| Numbers "two" and "ten" | Repeating aloud | Two words per second | Unspecified | Written | AS (E1); backward counting (E2) | Change-detection task (visual) | WM capacity | Colored squares | Unspecified | Morey et al. | 2015 |
| Numbers 2001-2005 (counting) | Repeating aloud | Encouraged to have a constant rate | Experimenter | Unspecified | AS, no AS control condition, spatial tapping (non-dominant hand) on a keyboard (numbers 1,2,3,6,5,4) | Problem solving (verbal insight, spatial insight, verbal non-insight, spatial non-insight) | Solution score | Problems | HE | Salmon-Mordekovich and Leikin | 2023 |
| Strings of four letters "ABCD" or "WXYZ", strings of four numbers "1234" or "6789" | Unspecified | 3-4 characters per second | Experimenter + audio recording | Written | AS, no AS control condition (E1, E2) | WMT (visual) | Stimulus-onset asynchrony | Colored squares (visually) | Unspecified (likely EN) | Woodman | 2021 |
| Syllable "ba, ba, ba" | Repeating aloud | Unspecified | Unspecified | Unspecified | AS, no control condition (E6a-b) | Visual imagery task | Response times | Colors and shapes | Unspecified | Cochrane et al. | 2019 |
| Syllable "ba" | Repeating aloud | Unspecified | Unspecified | Written | AS, labeling condition, experimental condition as secondary tasks (E1-E3) | Color fidelity task, visual WMT | Recall error | Objects (visually) | DE | Overkott and Souza | 2022 |
| Syllable "ba" | Unspecified | Unspecified | Online, not monitored | Unspecified | AS, no control condition (E1) | WMT | Proportion of correct responses, RT | Colored shapes (visually) | DE | Souza | 2025 |
| Syllable "bababa" | Unspecified | Continuously | Unspecified | Written | AS, no control condition (E2, E3a-b) | Serial recall task (E2: Continuous reproduction task; E3a: oral recall; E3b: typed recall) | Recall performance | Colors (E2), words | DE | Souza et al. | 2018 |
| Syllable "da" | Repeating aloud | 2 Hz | Unspecified | Unspecified | AS, no control condition (E4a-b, E5b) | Visual short-term memory task | Recall accuracy | Color patches (visually) | JA | Ueda et al. | 2023 |
| Syllable "la" | Repeating aloud | Unspecified | Unspecified | Unspecified | AS, no AS control condition (E2, E3) | Recognising speech in noise | Accuracy | Sentences (auditorily) | EN, ES | Gleason and Francis | 2024 |
| Syllable "za" | Unspecified | Every second | Unspecified | Auditory | AS, no control condition (E1-E5) | Change detection task (visual + tactile) | Reaction times, recall items held in memory | Array of reeds, CRT | JA | Yoshida et al. | 2015 |
| Syllables "ba bi bou" | Repeating aloud | Unspecified | Unspecified | Unspecified | AS, no control condition (E2) | WMT | Percentage of correct responses | Letters (visually) | Unspecified (likely DE) | Belletier et al. | 2021 |
| Syllables "ba bi bu" | Repeating aloud | 0.5 second per syllable | Audio-recording | Written | AS, no control condition (E3) | WMT | Proportion of correct answers | Digits, consonants, nouns (visually) | Unspecified (likely DE) | Oberauer | 2022 |
| Syllables "babebibobu" | Repeating aloud | Unspecified | Unspecified | Unspecified | AS, no control condition (E4) | WMT (visual) | Angular error in the color wheel | Colored squares (visually) | Unspecified (likely FR) | Kowialiewski et al. | 2022 |
| Syllables "babebibobu" | Repeating aloud | Unspecified | Unspecified | Unspecified | AS; no control condition (E1, E2) | Span task | Reaction times, recall performance | Series of letters, images | FR | Plancher et al. | 2019 |
| Syllables "babebibobu" | Repeating aloud | Unspecified | Unspecified | Unspecified | AS, no control condition (E1, E2) | Span WMT (during emotional modulation) | Correct serial recall | FR consonants | Unspecified (likely FR) | Chainay et al. | 2023 |
| Syllables "BaBiBo" | Unspecified | Unspecified | Unspecified | Unspecified | AS, no control condition | Verbal description task in two conditions (non-verbal or verbal) | Frequencies of correct responses, error types | Black-and-white short video clips of a human agent | EN (n = 41), FR (n = 44) | Engemann et al. | 2015 |
| Syllables "babibou" | Repeating aloud | 1Hz (one syllable per second) | Experimenter | Unspecified | AS, no control condition (E3, E4) | WMT (span task) | Accuracy on memory | Letters and numbers (visually) | Unspecified (likely FR) | Barrouillet et al. | 2024 |
| Syllables "babibou" | Unspecified | Unspecified | Unspecified | Unspecified | AS, no control condition (E1, E2) | WMT, long term memory | Strategy accuracy (d-prime) | Three word-pairs (visually) | Unspecified (likely DE) | Bhanap et al. | 2025 |
| Syllables "des-die-das" | Unspecified | Continuously (E1-E6) | Unspecified | Unspecified | AS, no control condition (E1-E6) | Recognition task (E1, E2:); Continuous reproduction task (E3-E6) | Proportion of correct responses (E1, E2); precision, target recall, non target recall, guessing (E3) ; precision, target recall, probabilities of errors (E4, E5); precision, of target recall, non target recall, guessing (E6) | Colored squares | Unspecified (likely DE) | Souza et al. | 2016 |
| Three digits | Unspecified | Unspecified | Unspecified | Unspecified | AS, no control condition (E1-E3) | Change-detection task | Accuracy, A' | Colored dots and squares | Unspecified | Holt and Delvenne | 2015 |
| Three digits | Unspecified | Unspecified | Unspecified | Written (E2) | AS, no control condition (E2) | Visual search task | Reaction times, recall performance | Color cues | Unspecified | Wen et al. | 2018 |
| Three digits number | Repeating aloud | 3 digits per second | Experimenter | Written | AS, no control condition | Staircase and attention tasks | Proportion of correct answers | Dots (visually) | Unspecified (likely EN) | Gong et al. | 2022 |
| Three digits number (e.g. 753) | Repeating aloud | Not too slowly and not to leave any gaps between the repetitions | Experimenter | Written | AS, no AS control condition (E3) | Flanker task | Error percentage, reaction time | Colored squares (visually) | Unspecified (likely TR) | Gunduz and Ozkan Ceylan | 2025 |
| Three letters (randomly chosen per trial) | Repeating aloud | One-two letters per second | Experimenter | Written | AS, no control condition (E1b, E2) | WMT (objects recall) | Recall performance | Color shapes (visually) | Unspecified (likely EN) | Schneegans et al. | 2023 |
| Three number digits | Repeating aloud | 3 digits per second | Unspecified | Written | AS, no control condition (E1, E2) | WMT (visual) | Visual WM accuracy | Visual objects | Unspecified (likely ZH) | Sun et al. | 2023 |
| Three-digit number | Repeating aloud (E1-E3) | Without pause between successive repetitions | Unspecified | Written | AS, no control condition (E1-E3) | WMT (visual) | Response time | Color-shape combinations | Unspecified | Geigerman et al. | 2016 |
| Two digit number | Repeating aloud | Unspecified | Unspecified | Written | AS, counting aloud (E1-E3) | Probed recall of a series of objects | Recall accuracy (E1, E2); recall accuracy, counting performance (E3) | Colored shapes | Unspecified | Hu et al. | 2016 |
| Two digits | Recite (unspecified; E1) | Continuously | Unspecified | Written | AS, no AS control condition (E1-E3) | PRD-manipulation (change detection task) | Visual memory capacity | Body movements, numbers (E1); nonsense shapes, animal images (E3) | Unspecified | Zhang et al. | 2019 |
| Two digits (E1a,c); Four digits (E1b) | Silently repeat in mind (E1a,c); Half of the blocks retained silent rehearsal, while the other half involved vocal rehearsal (1b) | Unspecified | Unspecified | Unspecified | AS, no control condition (E1a-c) | WMT (visual) | WM capacity (E1a-c) | Colors, real-word items | Unspecified | Quirk et al. | 2020 |
| Two digits 1-9 | Repeating aloud | Unspecified | Unspecified | Written | AS, no control condition (E1-E2) | Visual search task | Accuracy, reaction times | Colored cues | Unspecified | Carlisle and Woodman | 2019 |
| Two digits 20-99 | Repeat (no further specified) | Unspecified | Unspecified | Written | AS, backward counting (E6) | Integral-feature binding task | Accuracy (E6) | Rectangles | Unspecified | Wan et al. | 2020 |
| Two digits numbers | Repeating aloud | Self-paced | Unspecified | Written | AS, no control condition (E1, E2) | Working memory capacity (WMC) of gesture-command associations | Working memory capacities | Gestures (visually) | Unspecified (likely EN) | Gao et al. | 2023 |
| Two numbers | Repeating aloud | Unspecified | Unspecified | Written | AS, no control condition (E3) | Change-detection task | Response time, accuracy | Arrays of colored lines | Unspecified (likely EN) | Agauas et al. | 2020 |
| Two numbers 1-9 | Repeating aloud | Unspecified | Unspecified | Written | AS, no control condition | WMT (visual) | Memory capacity (k) | Animal silhouettes (visually) | Unspecified | Sun et al. | 2021 |
| Two one-syllable digits "1, 2, 3, 4, 5, 6, 8, 9" | Repeating aloud | Minimum four articulations every trial | Unspecified | Written ( | AS, no control condition (E3) | Change-detection task | Global precedence effect, feature-to-level bindings, feature-to-feature bindings, false alarm rate | Spatial frequency gratings, Navon figures | Unspecified | Ericson et al. | 2016 |
| Two sequential digits from 1-9 | Repeating aloud; subvocally | Unspecified | Unspecified | Auditory and Written | AS, no control condition (E1a-b, E2) | WMT (spatial); Visual search task | Accuracy, reaction times, recognition performance | Letters, squares | Unspecified | Annac et al. | 2019 |
| Two syllables, for example "badabada" | Same volume as the audio | 1 syllable per second | Unspecified | Auditory | AS, no control condition (E1-E3) | Span Task | Mean memory span, range of span, % correct responses as a function of gaze direction | Matrices of clusters, lines or geometric figures (E1, E2); Japanese symbols (E3) | Unspecified | Carlei and Kerzel | 2015 |
| Two-digit numbers 10-99 | Reading aloud | Unspecified | Unspecified | Written | AS, no control condition (E2) | Free recall task | Recall performance | Lists of words | Unspecified (likely EN) | Ecker et al. | 2015 |
| Word "Bla" | Repeating aloud | Unspecified | Unspecified | Written | AS, counting forward by 2 or 3 as secondary tasks (E2) | Contingency learning task | Probability of detecting a positive contingency | Colored shapes (visually) | Unspecified | Jozefowiez and Miller | 2024 |
| Word "Coca-cola" | Repeat (no further specified) | Repeat (no further specified) | Once per second | Unspecified | AS, no control condition (E1-E3) | Change-detection task | Hit rates | Shapes, letters, objects | Unspecified (likely acoustic) | Udale et al. | 2018 |
| Word "Coca-cola" | Unspecified | Unspecified | Unspecified | Unspecified | AS, no control condition (E1-E5) | Change detection task | Response times, hits | Colored items | Unspecified (likely EN) | Udale et al. | 2018 |
| Word "Coca-Cola" | Unspecified | Unspecified | Unspecified | Unspecified | AS, no control condition (E1a-b, E2) | WMT (visual) | Accuracy | Colored shapes (visually) | Unspecified | Xie and Wei | 2023 |
| Word "Cola" | Unspecified | Unspecified | Experimenter | Unspecified | AS, no control condition | WMT (visual, Memory updating task) | Accuracy and Reaction times | Colored rectangles (visually) | Unspecified | Lin et al. | 2021 |
| Word "mathématique" | Repeating aloud | Three words every two seconds | Unspecified | Unspecified | AS, no control condition (E2b) | Serial recall task | Recall performance (E2b) | Lists of words | Unspecified | Poirier et al. | 2015 |
| Word "the" | Unspecified | Unspecified | Unspecified | Unspecified | AS, no control condition (E4) | Episodic memory | Response time, immediate recall, delayed recall | Words (visually) | EN | Loaiza et al. | 2023 |
| Word "the" | Repeating aloud | Unspecified | Experimenter | Unspecified | AS, no control condition (E1-E4) | WMT | Memory strength (d-prime) | 6 real-world objects or 6 colored dots (visually) | Unspecified (likely EN) | Brady and Störmer | 2022 |
| Word "the" | Unspecified | Unspecified | Unspecified | Unspecified | AS, no control condition | WMT (visual, episodic and non-episodic memory task) | Performance | Coins | EN | Craig et al. | 2016 |
| Word "the" | Repeating aloud | Unspecified | Unspecified | Unspecified | AS, no control condition (E1-E5) | WMT, color memory task, letter detection task, single-object tracking task | Response error (in degrees) | Dots (visually), letters (visually), color dots + five points stars | Unspecified | Li and Li | 2021 |
| Word "the" | Verbally repeating (no volume specification) | ~2-3 repetitions per second | Unspecified | Written | AS, backward counting (E1-E3) | Change-detection task | Sensitivity, hits, false alarms | Colored shaped | Unspecified (likely EN) | Bocincova et al. | 2017 |
| Word "the" | Unspecified | Unspecified | Unspecified | Unspecified | AS, no control condition (E1-E3) | Recognition task (auditory, visual) | Recognition performance | Semantically related words | EN | Olszewska et al. | 2015 |
| Word "the" | Repeating aloud | Once a second | Unspecified | Unspecified | AS, no AS control condition (E1-E2) | Recognition task | Accuracy | Liquids | Unspecified | Daniel and Katz | 2018 |
| Word "the" | Repeating aloud | Unspecified | Unspecified | Written | AS, no AS control condition (E1, E2) | Serial recall task | Recall performance | Words, non-words (E1), non-words (E2) | EN | Ritchie et al. | 2015 |
| Word "the" | Unspecified | Unspecified | Unspecified | Unspecified | AS, no control condition (E1, E2) | Short term memory task, serial recall task | Words correctly recalled | Three phonemes long words (auditorily) | EN | James and Roodenrys | 2023 |
| Word "the" | Repeating (no further specified) | 3Hz rate | Unspecified | Unspecified | AS (replication study E1) | WMT (quantity vs quality) | Distribution of responses error, guess rate, response precision (E1) | Set of colors | Unspecified (likely EN) | Fougnie et al. | 2016 |
| Word "the" | Unspecified | Twice per second | Unspecified | Unspecified | AS, no control condition (E2) | Recognition task | Confidence of response, proportion of old responses | Japanese Kanji words | Kanji | Nishiyama et al. | 2017 |
| Word "the" | Repeating aloud | 4Hz | Unspecified | Unspecified | AS, no control condition (E4) | WMT (auditory, visual) | Performance | Bird calls (auditory task), position of white dots (visual task) | Unspecified | Fougnie et al. | 2015 |
| Words "mamma mia" | Unspecified | Unspecified | Unspecified | Unspecified | Verbal rehearsal | WMT (Web test reliability) | Distribution of samples and outliers | Series of letters (visually) | Unspecified | Uittenhove, et al. | 2023 |

**References**

Agauas, S. J., Jacoby, M., and Thomas, L. E., 2020. Near-hand effects are robust: Three OSF pre-registered replications of visual biases in perihand space. Visual Cognition, 28(3), 192–204. <https://doi.org/10.1080/13506285.2020.1751763>

Allen, R. J., Baddeley, A. D., and Hitch, G. J., 2017. Executive and perceptual distraction in visual working memory. Journal of Experimental Psychology: Human Perception and Performance, 43(9), 1677–1693. https://doi.org/[10.1037/xhp0000413](https://doi.org/10.1037/xhp0000413)

Allen, R. J., Castellà, J., Ueno, T., and Hitch, G. J., 2015. What does visual suffix interference tell us about spatial location in working memory? Memory & Cognition, 43(1), 133–142. <https://doi.org/10.3758/s13421-014-0448-4>

Annac, E., Zang, X., Müller, H. J., and Geyer, T., 2019. A secondary task is not always costly: Context‐based guidance of visual search survives interference from a demanding working memory task. British Journal of Psychology, 110(2), 381–399. <https://doi.org/10.1111/bjop.12346>^[[1]](#footnote-1)^

Barrouillet, P., Camos, V., Pougeon, J., Beaudet, J., Croizet, P., and Belletier, C., 2024. Human cognitive system privileges processing over short-term storage: Asymmetry in working memory limitations. Journal of Experimental Psychology: Learning, Memory, and Cognition. Advance online publication. <https://doi.org/10.1037/xlm0001362>

Belletier, C., Camos, V., and Barrouillet, P., 2021. Is the cognitive system much more robust than anticipated? Dual-task costs and residuals in working memory. Journal of ^[[2]](#footnote-2)^Experimental Psychology: Learning, Memory, and Cognition, 47(3), 498–517. https://doi.org/[10.1037/xlm0000961](https://doi.org/10.1037/xlm0000961)

Bhanap, R., Bartsch, L. M., and Rosner, A., 2025. Tracking reactivation of location information during memory strategies: Insights from eye movements. Journal of Cognition, 8(1), Article 38. https://[10.5334/joc.449](https://doi.org/10.5334/joc.449)

Blalock, L. D., 2015. Stimulus familiarity improves consolidation of visual working memory representations. Attention, Perception, & Psychophysics, 77(4), 1143–1158. <https://doi.org/10.3758/s13414-014-0823-z>

Bocincova, A., van Lamsweerde, A. E., and Johnson, J. S., 2017. The role of top-down suppression in mitigating the disruptive effects of task-irrelevant feature changes in visual working memory. Memory & Cognition, 45(8), 1411–1422. https://doi.org/10.3758/s13421-017-0738-8

Carlei, C., and Kerzel, D., 2015. The effect of gaze direction on the different components of visuo-spatial short-term memory. Laterality: Asymmetries of Body, Brain and Cognition, 20(6), 738–754. <https://doi.org/10.1080/1357650X.2015.1047380>

Carlisle, N. B., and Woodman, G. F., 2019. Quantifying the attentional impact of working memory matching targets and distractors. Visual Cognition, 27(5–8), 452–466. <https://doi.org/10.1080/13506285.2019.1634172>

Chainay, H., Ceresetti, R., Pierre-Charles, C., and Plancher, G., 2023. Modulation of maintenance and processing in working memory by negative emotions. Memory & Cognition, 51(8), 1774–1784. https://doi.org/[10.3758/s13421-023-01428-0](https://doi.org/10.3758/s13421-023-01428-0)

ochrane, B. A., Siddhpuria, S., and Milliken, B., 2019. Cueing color imagery: A critical analysis of imagery-perception congruency effects. Journal of Experimental Psychology: Learning, Memory, and Cognition, 45(8), 1410–1421. [https://doi.org/10.1037/xlm0000653](https://psycnet.apa.org/doi/10.1037/xlm0000653)

Craig, M., Butterworth, K., Nilsson, J., Hamilton, C. J., Gallagher, P., and Smulders, T. V., 2016. How does intentionality of encoding affect memory for episodic information? Learning & Memory, 23(11), 648–659. <https://doi.org/10.1101/lm.041491.115>

Daniel, T. A., and Katz, J. S., 2018. Primacy and recency effects for taste. Journal of Experimental Psychology: Learning, Memory, and Cognition, 44(3), 399–405. <https://doi.org/10.1037/xlm0000437>

Drew, T., Boettcher, S. E. P., and Wolfe, J. M., 2016. Searching while loaded: Visual working memory does not interfere with hybrid search efficiency but hybrid search uses working memory capacity. Psychonomic Bulletin & Review, 23(1), 201–212. <https://doi.org/10.3758/s13423-015-0874-8>

Dube, B., Basciano, A., Emrich, S. M., and Ferber, S., 2016. Visual working memory simultaneously guides facilitation and inhibition during visual search. Attention, Perception, & Psychophysics, 78(4), 1232–1244. https://doi.org/10.3758/s13414-016-1105-8

Ecker, U. K., Brown, G. D., and Lewandowsky, S., 2015. Memory without consolidation: Temporal distinctiveness explains retroactive interference. Cognitive Science, 39(7), 1570–1593. <https://doi.org/10.1111/cogs.12214>

Engemann, H., Hendriks, H., Hickmann, M., and Vitu, F., 2015. How language impacts memory of motion events in English and French. Cognitive Processing, 16(Suppl 1), 209–213. <https://doi.org/10.1007/s10339-015-0696-7>

Ericson, J. M., Beck, M. R., and van Lamsweerde, A. E., 2016. Binding global and local object features in visual working memory. Attention, Perception, & Psychophysics, 78(1), 94–106. https://doi.org/10.3758/s13414-015-1008-0

Fan, L., Sun, M., Xu, M., Li, Z., Diao, L., and Zhang, X., 2019. Multiple representations in visual working memory simultaneously guide attention: The type of memory-matching representation matters. Acta Psychologica, 192, 126–137. <https://doi.org/10.1016/j.actpsy.2018.11.005>

Ferreira, H. C. P., and Galera, C., 2016. The incidental binding of color and shape is insensitive to the perceptual load. Psicologia: Reflexão e Crítica, 29(1), 11. https://doi.org/10.1186/s41155-016-0012-1

Fougnie, D., Cormiea, S. M., Kanabar, A., and Alvarez, G. A., 2016. Strategic trade-offs between quantity and quality in working memory. Journal of Experimental Psychology: Human Perception and Performance, 42(8), 1231–1240. https://doi.org/10.1037/xhp0000211

Fougnie, D., Zughni, S., Godwin, D., and Marois, R., 2015. Working memory storage is intrinsically domain specific. Journal of Experimental Psychology: General, 144(1), 30–47. https://doi.org/10.1037/a0038211

Gao, Q., Ma, Z., Gu, Q., Li, J., and Gao, Z., 2023. Working memory capacity for gesture-command associations in gestural interaction. International Journal of Human–Computer Interaction, 39(15), 3045–3056. <https://doi.org/10.1080/10447318.2022.2091213>.

Geigerman, S., Verhaeghen, P., and Cerella, J., 2016. To bind or not to bind, that's the wrong question: Features and objects coexist in visual short-term memory. Acta Psychologica, 167, 45–51. https://doi.org/10.1016/j.actpsy.2016.04.004

Gleason, L. J., and Francis, W. S., 2024. Mechanisms of long-term repetition priming in recognising speech in noise. Memory, 32(2), 237–251. <https://doi.org/10.1080/09658211.2024.2305872>

Gong, M., Chen, Y., and Liu, T., 2022. Preparatory attention to visual features primarily relies on non-sensory representation. Scientific Reports, 12(1), Article 21726. https://doi.org/10.1038/s41598-022-26104-2

Guitard, D., Gabel, A. J., Saint-Aubin, J., Surprenant, A. M., and Neath, I., 2018. Word length, set size, and lexical factors: Re-examining what causes the word length effect. Journal of Experimental Psychology: Learning, Memory, and Cognition, 44(11), 1824–1844. [https://doi.org/10.1037/xlm0000551](https://psycnet.apa.org/doi/10.1037/xlm0000551)

Gunduz, H., and Ozkan Ceylan, A., 2025. Load effect of visual working memory on distractor interference: An investigation with two replication experiments. Memory & Cognition, 53(5), 832–852. <https://doi.org/10.3758/s13421-024-01610-y>

Hakim, N., deBettencourt, M. T., Awh, E., and Vogel, E. K., 2020. Attention fluctuations impact ongoing maintenance of information in working memory. Psychonomic Bulletin & Review, 27(6), 1269–1278. <https://doi.org/10.3758/s13423-020-01790-z>

Hu, Y., Allen, R. J., Baddeley, A. D., and Hitch, G. J., 2016. Executive control of stimulus-driven and goal-directed attention in visual working memory. Attention, Perception, & Psychophysics, 78(7), 2164–2175. <https://doi.org/10.3758/s13414-016-1106-7>

Hurlstone, M. J., and Hitch, G. J., 2018. How is the serial order of a visual sequence represented? Insights from transposition latencies. Journal of Experimental Psychology: Learning, Memory, and Cognition, 44(2), 167–192. https://doi.org/10.1037/xlm0000440

James, T., and Roodenrys, S., 2023. Exploring the necessary conditions for phonological interference in serial recall. Memory, 31(7), 891–904. <https://doi.org/10.1080/09658211.2023.2200587>

Ji, E., Lee, K. M., and Kim, M. S., 2017. Independent operation of implicit working memory under cognitive load. Consciousness and Cognition, 55, 214–222. https://doi.org/10.1016/j.concog.2017.08.014

Jozefowiez, J., and Miller, R. R., 2024. Cue duration and trial spacing effects in contingency assessment in the streaming procedure with humans. Journal of Experimental Psychology: Animal Learning and Cognition, 50(2), 99–112. https://doi.org/[10.1037/xan0000376](https://doi.org/10.1037/xan0000376).

Kiverstein, J. (2020). Embodied cognition and the neural reuse hypothesis. In Current controversies in philosophy of cognitive science (pp. 87-107). Routledge.

Kowialiewski, B., Lemaire, B., and Portrat, S., 2022. Between-item similarity frees up working memory resources through compression: A domain-general property. Journal of Experimental Psychology: General, 151(11), 2641–2660. https://doi.org/[10.1037/xge0001235](https://doi.org/10.1037/xge0001235)

Lagacé, S., and Guérard, K., 2015. When motor congruency modulates immediate memory for objects. Acta Psychologica, 157, 65–73. <https://doi.org/10.1016/j.actpsy.2015.02.009>

Li, Z., and Li, Z., 2021. Dual-task costs in memory recall precision reflect shared representational space. Journal of Experimental Psychology: Human Perception and Performance, 47(3), 460–474. https://doi.org/[10.1037/xhp0000894](https://doi.org/10.1037/xhp0000894)

Lin, Y. T., Sasin, E., and Fougnie, D., 2021. Selection in working memory is resource-demanding: Concurrent task effects on the retro-cue effect. Attention, Perception, & Psychophysics, 83(5), 1600–1612. <https://doi.org/10.3758/s13414-020-02239-0>

Loaiza, V. M., Oftinger, A. L., and Camos, V., 2023. How does working memory promote traces in episodic memory? Journal of Cognition, 6(1), Article 4. https://doi.org/[10.5334/joc.245](https://doi.org/10.5334/joc.245)

Matsukura, M., and Vecera, S. P., 2015. Selection of multiple cued items is possible during visual short-term memory maintenance. Attention, Perception, & Psychophysics, 77(5), 1625–1646. https://doi.org/10.3758/s13414-015-0836-2

Morey, C. C., 2019. Perceptual grouping boosts visual working memory capacity and reduces effort during retention. British Journal of Psychology, 110(2), 306–327. https://doi.org/10.1111/bjop.12355

Morey, C. C., Cong, Y., Zheng, Y., Price, M., and Morey, R. D., 2015. The color-sharing bonus: Roles of perceptual organization and attentive processes in visual working memory. Archives of Scientific Psychology, 3(1), 18–29. <https://doi.org/10.1037/arc0000014>

Nishiyama, R., Hirano, T., and Ukita, J., 2017. Usage of semantic representations in recognition memory. Memory, 25(10), 1412–1424. https://doi.org/10.1080/09658211.2017.1310252

Norris, D., Hall, J., Butterfield, S., and Page, M. P. A., 2019. The effect of processing load on loss of information from short-term memory. Memory, 27(2), 192–197. https://doi.org/10.1080/09658211.2018.1497661

Olszewska, J. M., Reuter-Lorenz, P. A., Munier, E., and Bendler, S. A., 2015. Misremembering what you see or hear: Dissociable effects of modality on short- and long-term false recognition. Journal of Experimental Psychology: Learning, Memory, and Cognition, 41(5), 1316–1325. https://doi.org/10.1037/xlm0000115

Overkott, C., and Souza, A. S., 2022. Verbal descriptions improve visual working memory but have limited impact on visual long-term memory. Journal of Experimental Psychology: General, 151(2), 321–337. https://doi.org/[10.1037/xge0001084](https://doi.org/10.1037/xge0001084)

Pan, Y., and Zhang, X., 2020. Visual working memory enhances target discrimination accuracy with single-item displays. Attention, Perception, & Psychophysics, 82(7), 3005–3012. <https://doi.org/10.3758/s13414-020-02041-y>

Pan, Y., Zhang, Z., and Zuo, W., 2019. The contents of visual working memory delay the perceived offset of matching visual stimuli. Acta Psychologica, 201, 102954. <https://doi.org/10.1016/j.actpsy.2019.102954>

Plancher, G., Massol, S., Dorel, T., and Chainay, H., 2019. Effect of negative emotional content on attentional maintenance in working memory. Cognition and Emotion, 33(7), 1489–1496. <https://doi.org/10.1080/02699931.2018.1561420>

Poirier, M., Saint-Aubin, J., Mair, A., and Guérard, K., 2015. Order recall in verbal short-term memory: The role of semantic networks. Memory & Cognition, 43(4), 489–499. https://doi.org/10.3758/s13421-014-0470-6

Quirk, C., Adam, K. C., and Vogel, E. K., 2020. No evidence for an object working memory capacity benefit with extended viewing time. eNeuro, 7(5). https://doi.org/10.1523/ENEURO.0150-20.2020

Ritchie, G., Tolan, G. A., and Tehan, G., 2015. Redintegration, task difficulty, and immediate serial recall tasks. Canadian Journal of Experimental Psychology / Revue canadienne de psychologie expérimentale, 69(1), 54–63. https://doi.org/10.1037/cep0000031

Romani, C., McAlpine, S., Olson, A., Tsouknida, E., and Martin, R., 2005. Length, lexicality, and articulatory suppression in immediate recall: Evidence against the articulatory loop. Journal of Memory and Language, 52(3), pp.398-415. <https://doi.org/10.1016/j.jml.2005.01.005>

Russo, R., and Grammatopoulou, N., 2003. Word length and articulatory suppression affect short-term and long-term recall tasks. Memory & Cognition, 31(5), pp.728-737. <https://doi.org/10.3758/BF03196111>

Salmon-Mordekovich, N., and Leikin, M., 2023. Insight problem solving is not that special, but business is not quite “as usual”: Typical versus exceptional problem-solving strategies. Psychological Research, 87(6), 1995–2009. <https://doi.org/10.1007/s00426-022-01786-5>

Schneegans, S., McMaster, J., and Bays, P. M., 2023. Role of time in binding features in visual working memory. Psychological Review, 130(1), 137–165. https://doi.org/[10.1037/rev0000331](https://doi.org/10.1037/rev0000331)

Sense, F., Morey, C. C., Prince, M., Frankish, C., and van der Maas, H. L. J., 2017. Opportunity for verbalization does not improve visual change detection performance: A state-trace analysis. *Behavior Research Methods*, *49*(3), 853–862. <https://doi.org/10.3758/s13428-016-0741-1>

Siedenburg, K., and McAdams, S., 2017. The role of long-term familiarity and attentional maintenance in short-term memory for timbre. *Memory*, *25*(4), 550–564. <https://doi.org/10.1080/09658211.2016.1197945>

Soemer, A., and Saito, S., 2015. Maintenance of auditory-nonverbal information in working memory. *Psychonomic Bulletin & Review*, *22*(6), 1777–1783. https://doi.org/10.3758/s13423-015-0854-z

Souza, A. S., 2025. Refreshing multi-feature objects in visual working memory. *Journal of Cognition, 8*(1), Article 49. https://doi.org/[10.5334/joc.464](https://doi.org/10.5334/joc.464)

Souza, A. S., Rerko, L., and Oberauer, K., 2016. Getting more from visual working memory: Retro-cues enhance retrieval and protect from visual interference. *Journal of Experimental Psychology: Human Perception and Performance*, *42*(6), 890–910. <https://doi.org/10.1037/xhp0000192>

Souza, A. S., Vergauwe, E., and Oberauer, K., 2018. Where to attend next: Guiding refreshing of visual, spatial, and verbal representations in working memory. *Annals of the New York Academy of Sciences*, *1424*(1), 76–90. https://doi.org/10.1111/nyas.13621

Sun, N., Han, H., Hao, J., and Lyu, P., 2025. Semantic relationships among objects reduce the attention required for inter-item binding in working memory. *BMC Psychology, 13*(1), Article 13. https://doi.org/10.1186/s40359-025-03099-9

Sun, Y., Song, J., Xin, X., Ding, X., and Li, S., 2021. Same-category advantage on the capacity of visual working memory. *Acta Psychologica Sinica, 53*(11), 1189–1202. <https://doi.org/10.3724/SP.J.1041.2021.01189>

Sun, Y., Wang, L., Sun, N., and Li, S., 2023. Modulation of similarity on the distraction resistance of visual working memory representation. *Psychophysiology, 60*(1), Article e14153. <https://doi.org/10.1111/psyp.14153>

Udale, R., Farrell, S., and Kent, C., 2018. No evidence of binding items to spatial configuration representations in visual working memory. *Memory & Cognition*, *46*(6), 955–968. https://doi.org/10.3758/s13421-018-0814-8

Udale, R., Farrell, S., and Kent, C., 2018. Task demands determine comparison strategy in whole probe change detection. *Journal of Experimental Psychology: Human Perception and Performance*, *44*(5), 778–796. https://doi.org/10.1037/xhp0000490

Ueda, Y., Huang, T. R., Shen, Z., Sakata, C., Yeh, S. L., and Saito, S., 2023. Sequential processing facilitates Hebb repetition learning in visuospatial domains. *Journal of Experimental Psychology: General, 152*(9), 2559–2575. https://doi.org/[10.1037/xge0001406](https://doi.org/10.1037/xge0001406)

Uittenhove, K., Jeanneret, S., and Vergauwe, E., 2023. From lab-testing to web-testing in cognitive research: Who you test is more important than how you test. *Journal of Cognition, 6*(1), Article 13. https://doi.org/[10.5334/joc.259](https://doi.org/10.5334/joc.259)

Valenti, L., and Galera, C., 2020. Dynamic visual noise has the same effect on visual memory and visual imagery tasks. *Psychology & Neuroscience*, *13*(1), 114–125. https://doi.org/10.1037/pne0000183

Wen, W., Hou, Y., and Li, S., 2018. Memory guidance in distractor suppression is governed by the availability of cognitive control. *Attention, Perception, & Psychophysics*, *80*(5), 1157–1168. https://doi.org/10.3758/s13414-018-1511-1

Woodman, G. F., 2021. Spatial location is filtered out of visual working memory representations when task irrelevant, just like other features. *Attention, Perception, & Psychophysics, 83*(4), 1391–1396. <https://doi.org/10.3758/s13414-021-02263-8>

Xie, T., and Wei, Y., 2023. Effects of temporal order and relative location on distractor interference in visual working memory. *Current Psychology, 42*(35), 31035–31047. https://doi.org/10.1007/s12144-022-04079-7

Yoshida, T., Yamaguchi, A., Tsutsui, H., and Watanabe, K., 2015. Tactile search for change has less memory than visual search for change. *Attention, Perception, & Psychophysics*, *77*(4), 1200–1211. <https://doi.org/10.3758/s13414-014-0829-6>

Zhang, L., Qiao, L., Xu, M., Che, X., Diao, L., Yuan, S., et al., 2019. Role of personal relative deprivation in promoting working memory capacity for neutral social information: Facial expressions and body motions. *Personality and Individual Differences*, *150*, 109464. https://doi.org/10.1016/j.paid.2019.06.007

1. [↑](#footnote-ref-1)
2. [↑](#footnote-ref-2)
